# Regulation of Pkc1 Hyper-Phosphorylation by Genotoxic Stress

**DOI:** 10.3390/jof7100874

**Published:** 2021-10-17

**Authors:** Li Liu, Jiri Veis, Wolfgang Reiter, Edwin Motari, Catherine E. Costello, John C. Samuelson, Gustav Ammerer, David E. Levin

**Affiliations:** 1 Department of Molecular and Cell Biology, Boston University Goldman School of Dental Medicine, Boston, MA 02118, USA; liul@bu.edu (L.L.); motari1@gmail.com (E.M.); jsamuels@bu.edu (J.C.S.); 2 Department of Biochemistry and Cell Biology, Max Perutz Labs, University of Vienna, 1030 Wien, Austria; jiri.veis@univie.ac.at (J.V.); reiterw6@univie.ac.at (W.R.); gustav.ammerer@univie.ac.at (G.A.); 3 Center for Medical Biochemistry, Max Perutz Laboratories, Medical University of Vienna, 1030 Wien, Austria; 4 Mass Spectrometry Facility, Max Perutz Laboratories, University of Vienna, 1030 Wien, Austria; 5 Department of Biochemistry, Center for Biomedical Mass Spectrometry, School of Medicine, Boston University, Boston, MA 02118, USA; cecmsms@bu.edu; 6 Department of Microbiology, Boston University School of Medicine, Boston, MA 02118, USA

**Keywords:** Hrr25, Mec1, Tel1, Pkc1, hydroxyurea, UV irradiation

## Abstract

The cell wall integrity (CWI) signaling pathway is best known for its roles in cell wall biogenesis. However, it is also thought to participate in the response to genotoxic stress. The stress-activated protein kinase Mpk1 (Slt2, is activated by DNA damaging agents through an intracellular mechanism that does not involve the activation of upstream components of the CWI pathway. Additional observations suggest that protein kinase C (Pkc1), the top kinase in the CWI signaling cascade, also has a role in the response to genotoxic stress that is independent of its recognized function in the activation of Mpk1. Pkc1 undergoes hyper-phosphorylation specifically in response to genotoxic stress; we have found that this requires the DNA damage checkpoint kinases Mec1 (Mitosis Entry Checkpoint) and Tel1 (TELomere maintenance), but not their effector kinases. We demonstrate that the casein kinase 1 (CK1) ortholog, Hrr25 (HO and Radiation Repair), previously implicated in the DNA damage transcriptional response, associates with Pkc1 under conditions of genotoxic stress. We also found that the induced association of Hrr25 with Pkc1 requires Mec1 and Tel1, and that Hrr25 catalytic activity is required for Pkc1-hyperphosphorylation, thereby delineating a pathway from the checkpoint kinases to Pkc1. We used SILAC mass spectrometry to identify three residues within Pkc1 the phosphorylation of which was stimulated by genotoxic stress. We mutated these residues as well as a collection of 13 phosphorylation sites within the regulatory domain of Pkc1 that fit the consensus for CK1 sites. Mutation of the 13 Pkc1 phosphorylation sites blocked hyper-phosphorylation and diminished *RNR3* (RiboNucleotide Reductase) basal expression and induction by genotoxic stress, suggesting that Pkc1 plays a role in the DNA damage transcriptional response.

## 1. Introduction

The cell wall integrity (CWI) signaling pathway of the budding yeast *Saccharomyces cerevisiae* has been well characterized with regard to its regulation by cell wall stress [1,2,3,4]. This pathway regulates biosynthesis of cell wall polymers, organization of the actin cytoskeleton, exocytosis, and the protein kinase C 1 (Pkc1)-mediated stress-activated protein kinase (SAPK) cascade through activation of the small GTPase, Rho1. The SAPK cascade is a linear pathway comprised of Pkc1, a MEKK (Bck1), a pair of redundant MEKs (Mkk1/2) and a SAPK (Mpk1/Slt2). The activation of Mpk1, in response to cell wall stress or hyper-activation of upstream pathway components, drives transcription in support of cell wall biogenesis [5,6,7,8,9] through the SRF-like transcription factor Rlm1 [10,11] and the cell cycle transcription factor SBF [12,13,14,15]. Moreover, loss-of-function mutants in the SAPK cascade display cell lysis defects that are suppressed by external osmotic support [2], highlighting the central role of this signaling pathway in the maintenance of cell wall integrity.

Although the CWI pathway is best understood for its essential role in maintaining the structural integrity of the cell wall during growth, morphogenesis, and in response to cell wall stressors, there is evidence that this pathway is also important for survival of genomic stress. Defects in any component of the CWI pathway, from the cell surface sensors to Mpk1, cause hyper-sensitivity to a variety of DNA damaging agents [16,17,18,19,20]. The CWI pathway SAPK Mpk1 is phosphorylated by the DNA damage checkpoint kinases Mec1 and Tel1 in response to treatment with methylmethane sulfonate (MMS) [21] or caffeine [22]. The Mec1 and Tel1 protein kinases are orthologs of mammalian ATR and ATM, respectively [23], and have overlapping but distinct functions in the maintenance of yeast genome integrity. Tel1 signals the presence of double-strand breaks specifically [24], whereas Mec1 signals the presence of a variety of DNA damage types, including double-strand breaks [25]. Mec1 and Tel1 phosphorylate and activate the checkpoint effector kinases Chk1 and Rad53, which mediate cell cycle arrest and gene expression in support of DNA repair [26,27,28,29]. 

In addition to phosphorylation of Mpk1, Soriano–Carot et al. [30] detected a DNA damage-induced hyper-phosphorylation of Pkc1. They suggested the existence of a reciprocal regulatory circuit in which Pkc1 was required to activate the DNA damage checkpoint and the DNA damage checkpoint kinase Tel1 was required for the phosphorylation-induced Pkc1 band-shift in response to DNA damage. However, we found that the DNA damage checkpoint is activated normally in *pkc1*Δ mutants from various strain backgrounds [31]. Additionally, there is a bifurcation of pathway outputs at Pkc1, first revealed as a 100-fold increase in mitotic recombination frequency in pkc1 mutants that is not observed in mutants of pathway components below Pkc1 [32]. This is significant because mitotic recombination is the principal mode of double-stranded break repair of DNA in yeast [33]. Moreover, Pkc1 phosphorylates and activates CTP synthetase directly [34], revealing a role in nucleotide metabolism. 

Finally, Mpk1 is activated by genotoxic stress through a pathway that does not require the activation of Pkc1 or the other protein kinases that function above Mpk1, but involves ubiquitination and degradation of Msg5, the protein phosphatase that normally maintains Mpk1 in a low activity state [31,35]. Intriguingly, Mpk1 activated in response to genotoxic stress does not drive cell wall stress transcription, suggesting that its activation in this context has a different function. These observations have led to the proposal that Pkc1 plays important roles in the response to genotoxic stress that are separate from its function in the activation of the CWI SAPK cascade [30], but these roles have not been elucidated. We hypothesize that the CWI pathway plays multiple unrecognized roles in the response to DNA damage—some driven by Mpk1, others by another pathway branch from Pkc1. In this study, we establish the pathway through which Pkc1 is hyper-phosphorylated in response to DNA damage and identify several sites within the Pkc1 regulatory domain whose phosphorylation is stimulated under conditions of genotoxic stress. Mutation of these sites impacts DNA damage-regulated gene expression.

## 2. Materials and Methods 

### 2.1. Strains, Growth Conditions, and Transformations

The *S. cerevisiae* strains used in this study were derived from the EG123 background [36], the RDK2669 background (M. Smolka), the W303 background (J.C. Igual), or the Research Genetics background BY4742 (Research Genetics, Inc.; Huntsville, AL, USA) and are listed in Table 1. 

Yeast cell cultures were grown in YPD (1% Bacto yeast extract, 2% Bacto Peptone, 2% glucose) or minimal selective medium, SD (0.67% Yeast nitrogen base, 2% glucose) supplemented with the appropriate nutrients to select for plasmids, which are listed in Table 2. Yeast cells were transformed according to Geitz et al. [38]. Sorbitol (0.5 M) was used to prevent cell lysis for pkc1∆ strains. Cell wall stress was induced by treatment with calcofluor white (CFW, 40 μg/mL; Millipore Sigma, Burlington, MA), or by heat shock at 39 °C. Genotoxic stress was induced by treatment with hydroxyurea (HU; 250 mM, except where indicated otherwise; Fisher Scientific, Waltham, MA) or ultraviolet light (UV; 150 J/m^2^ for Pkc1 band-shift, or 250 J/m^2^ for viability assay). UV irradiation was carried out using an Analytik Jena UVP Crosslinker (Fisher Scientific). Cultures for viability tests were pelleted and resuspended in phosphate-buffered saline (PBS), dispersed on the surface of an empty petri dish for irradiation prior to dilution and plating. Cultures for Pkc1 band-shift were incubated in YPD for an additional 2 h for recovery after irradiation. To inhibit Hrr25 catalytic activity, 5 μM or 10 μM of PP1 analog IV, PP1 analog II (1NM PP1), or PP1 analog (Millipore Sigma) were used in agar plates. For Hrr25 inhibition in culture, PP1 analog IV (20 μM) was added to cultures at the time of addition of HU and incubated for 4 h.

### 2.2. Chromosomal Deletions and Strain Construction

A *sml1*∆::*TRP1 mec1*∆::*HIS3 tel1*∆::*URA3* strain (DL4277) was generated as a meiotic segregant of DL3954 (*MAT***a**/α MBS115 *SML1*/*sml1*∆::*TRP1 MEC1*/*mec1*∆::*HIS3 TEL1*/*tel1*∆::*URA3*). An *sml1*∆::*TRP1 mec1*∆::*KanMX tel1*∆::*URA3* strain (DL4503) was created by homologous recombination of *mec1*∆::*KanMX* at the *MEC1* locus in an *sml1*∆::*TRP1 tel1*∆::*URA3* strain (DL3951). The *mec1*∆::*KanMX* allele was amplified by PCR from genomic DNA of yeast strain (DL3952) using Phusion high-fidelity DNA polymerase (Thermo Fisher Scientific, San Jose, CA). Transformants were selected for antibiotic G418 resistance and validated by genomic PCR across both integration junction sites. Endogenous tagging of Pkc1 with HTBeaq [43] was achieved by transformation of BY4741 with HB-tagging cassettes amplified from plasmid pWR268 [43], resulting in yeast strain JV826.

Plasmid-borne “gatekeeper” alleles of *HRR25* were tested for function initially in an *hrr25*Δ strain maintained with a plasmid-borne, *GAL1*-controlled *HRR25^degron^* (DL4290) [37], which is viable only in galactose-containing medium. Later experiments with these mutant alleles were conducted in an *hrr25*Δ strain without the *HRR25^degron^*. To create *hrr25*Δ::*HPHMX4* in W303 (DL4515), a plasmid bearing *HRR25-HA* (p3484; pRS425-*HRR25-HA*) was first introduced into DL4206 (*MAT***a** W303 *ade2 trp1 leu2 his3 ura3 can1*). The *HPHMX4* gene was amplified by PCR from plasmid p3064 (pAG32-*RGC2*) using primers with extensions homologous to the 5′ and 3′ ends of *HRR25* and used to transform DL4206 pRS425-*HRR25-HA* to hygromycin B resistance. Gene replacement was validated by PCR analysis across both integration junctions. The plasmid-borne *HRR25* in DL4515 was replaced with other alleles of *HRR25* carried on pRS313 or pRS423 using plasmid shuffle. 

### 2.3. Plasmid Construction and Mutagenesis

The *HRR25* gene was epitope-tagged on its C-terminus with 3xHA and expressed from its natural promoter. The promoter region of *HRR25* (from position −979) and its entire coding sequence was amplified from genomic yeast DNA (DL2772) by high-fidelity PCR polymerase (Phusion) using primers designed with a HindIII site (upstream) and a NotI site (downstream), and subcloned into pRS425-*3HA*-*ADH1^T^* (p3149) to generate pRS425-*HRR25-HA* (p3484). To create pRS425-*hrr25-*∆*404-HA* (p3538), the promoter region of *HRR25* (from position −979) and its C-terminally truncated coding sequence was amplified from pRS425-*HRR25-HA* (p3484) by high-fidelity PCR polymerase (Phusion) using primers designed with a HindIII site (upstream) and a NotI site (downstream) and subcloned into pRS425-*3HA-ADH1^T^* (p3149). 

To express HA-tagged *HRR25* from *HIS3*-marked plasmids pRS313-*3HA-ADH1^T^* (p3504) and pRS423-*3HA-ADH1^T^* (p3544), first required subcloning of the *3HA-ADH1^T^* sequence from pRS425-*3HA-ADH1^T^* (p3149) into pRS313 or pRS423 [39] at SmaI and SacI sites. Plasmid pRS313-*HRR25-HA* (p3545) was next created by subcloning a Sal1-Not1 fragment containing the promoter region of *HRR25* (from position −979) and its entire coding sequence from pRS425-*HRR25-HA* (p3484) into pRS313-*3HA-ADH1^T^* (p3504). Plasmid pRS313-*hrr25-*∆*404-H*A (p3546) was created similarly. Plasmid pRS423-*HRR25-HA* (p3547) was generated by subcloning the Sal1-Not1 fragment containing *HRR25* into pRS423-*3HA-ADH1^T^* (p3544).

The *HRR25* gene was also epitope-tagged on its C-terminus with GFP and expressed from its natural promoter. A PCR fragment containing the GFP coding sequence was amplified from pRS425-*GFP* (p1202) with primers designed with Sma1 and Not1 sites (upstream) and a SacII site (downstream) and cloned into pRS423 to create GFP-tagging vector pRS423-*GFP* (p3560). An Apa1-Not1 fragment containing the promoter region of *HRR25* (from position −979) and its entire coding sequence digested from pRS423-*HRR25-HA* (p3547) was subcloned into pRS423-*GFP* (p3560) at the Apa1-Not1 sites to generate pRS423-*HRR25-GFP* (p3562). Plasmid pRS423-*hrr25-*∆*404-GFP* (p3567) was similarly created from pRS313-*hrr25-*∆*404-HA* (p3546). 

Point mutations in *PKC1* were constructed initially in high copy plasmid YEp351-*PKC1-HA* (p813) for use in band-shift assays. However, for phenotypic analyses, we expressed mutant alleles of *PKC1-HA* from a centromeric plasmid. To create pRS314-*PKC1-HA*, *pkc1-3A-HA*, and *pkc1-S/T13A-HA*, *PKC1-HA* (from position -950) with its 3′ region (640 bp) were PCR amplified from YEp351-*PKC1-HA* (p813), YEp351-*pkc1-S3A-HA* (p3619) or YEp351-*pkc1-S/T13A-HA* (p3612), respectively, by PrimeSTAR, Max DNA Polymerase (Takara Bio USA, Mountainview, CA) using primers designed with a SalI site (upstream) and with a SacII site (downstream) and cloned into pRS314 (p118).

All point mutations in *HRR25* or *PKC1* were created using QuickChange or QuikChange Lightning Site-Directed Mutagenesis Kits (Agilent Technologies, Santa Clara) according to manufacturer’s instructions. All DNA sequences derived from PCR mutagenesis were confirmed by sequence across the entire gene.

### 2.4. Protein Extraction

Protein extraction was carried out as described previously for co-immunoprecipitation [44], or using the rapid boiling method [45] for direct immunoblot experiments. 

### 2.5. β-galactosidase Measurements

Measurements of β-galactosidase activity from *RNR3-lacZ* (p2947) expression experiments were conducted in triplicate and carried out as described in Zhao et al. [46].

### 2.6. Dephosphorylation Assay

Protein extracts were prepared from *sml1*∆ (DL3950) cells carrying PKC1-HA (p813), treated with 250 mM HU or untreated, by bead-beating in lysis buffer (10% glycerol, 20 mM Hepes pH 7.5, 150 mM NaCI, 0.5% triton X-100 and Mini protease inhibitor cocktail (Milipore Sigma), without phosphatase inhibitors, followed by centrifugation. The dephosphorylation reactions were performed using Lambda protein phosphatase (New England Biolabs, Ipswich, MA) according to manufacturer’s protocol. Briefly, 10 µg of protein extract was incubated with 400 units of Lambda protein phosphatase in Lambda protein phosphatase buffer for 30 min at 30 °C. Phosphatase reactions were stopped by adding an equal volume of 2× SDS sample buffer and the resulting samples were boiled for 3 min prior to SDS-PAGE and immunoblot analysis. 

### 2.7. Co-immunoprecipitation

To detect association of Pkc1 with Hrr25 in wild-type cells, a plasmid expressing Pkc1-HA under the control of its own promoter (p813) was co-transformed with plasmids expressing either GFP-Hrr25 (p3357) or GFP alone (p3358) into DL100. Transformants were grown to mid-log phase in selective medium and starved for methionine for two hours to induce expression of GFP-Hrr25 or GFP, which were expressed under the control of the *MET25* promoter. Cultures were then treated with 250 mM HU for 4 h. To test the effect of *MEC1* and *TEL1* loss on the association of Pkc1 with Hrr25, a plasmid expressing Hrr25-GFP under the control of its own promoter (p3562) was co-transformed with p813 into strains DL3950 *(sml1*Δ) and DL4503 (*sml1*Δ *mec1*∆ *tel1*∆). To test the association of Pkc1-HA with truncated Hrr25 (Hrr25-Δ404-GFP), plasmid p813 was transformed into strains bearing a chromosomal deletion of *HRR25* complemented by plasmids expressing either Hrr25-GFP (DL4541) or Hrr25-∆404-GFP (DL4542). Transformants were grown to mid-log phase in selective medium and treated with 250 mM HU for 4 h. Protein extracts were made as described previously [44]. Extracts (100 µg of protein) were incubated with 10µL of GFP-trap agarose beads (Chromotek, Munich, Germany) at 4 °C for 2 h and the samples were washed with IP buffer (50 mM Tris-HCl, pH 7.5, 150 mM NaCl, 0.5% Triton) three times and boiled in SDS-PAGE buffer. 

### 2.8. SDS-PAGE Electrophoresis and Immunoblot Analysis

Proteins were separated by SDS-PAGE (10% or 4–20% Mini-PROTEAN TGX gels, BioRad, Hercules, CA, USA) followed by immunoblot analysis using mouse monoclonal α-HA (BioLegend, San Diego, CA, USA) or α-GFP (Millipore Sigma) at a dilution of 1:10,000 and polyclonal rabbit α-Rad53 (Abcam) antibodies at a dilution of 1:2000 to detect Pkc1-HA or -GFP, Hrr25-HA or -GFP and Rad53, respectively. Secondary goat anti-mouse (Jackson ImmunoResearch, Westgrove, PA, USA) and donkey anti-rabbit (GE Healthcare, Chicago, IL, USA) antibodies were used at a dilution of 1:10,000. SDS-PAGE gels used to detect Pkc1 band-shifts were 10% and those used for Pkc1 association with Hrr25 were 4–20%. 

### 2.9. Mass Spectrometric Analysis of Pkc1 Co-Immunoprecipitates

Pkc1-GFP was expressed from plasmid p2062 (gift of M. Cyert) in strain DL100 and isolated using GFP-trap agarose. Pkc1-GFP immunoprecipitates were run on SDS-PAGE gels prior to in-gel digestion of the contents of the entire lane with trypsin. After desalting, LC-MS/MS was performed using a nanoAcquity ultra-performance liquid chromatography (UPLC) capillary system (Waters Corp., Milford, MA, USA), coupled to an LTQ-Orbitrap hybrid mass spectrometer (Thermo Fisher Scientific) equipped with a TriVersa NanoMate ion source (Advion, Ithaca, NY, USA). Sample concentration and desalting were performed online using a nanoAcquity UPLC trapping column (180 μm by 20 mm; packed with 5-μm, 100-Å-pore-size Symmetry C_18_ material; Waters Corp.) at a flow rate of 15 μL/min for 1 min. Separation was accomplished on a nanoAcquity UPLC capillary column (100 μm by 100 mm; packed with 1.7-μm, 130-Å-pore-size bridged ethyl hybrid [BEH] C_18_ material; Waters Corp.). A linear gradient of A and B buffers (buffer A, 3% ACN–0.1% formic acid [FA]; buffer B, 97% ACN–0.1% FA) from 7% to 45% buffer B over 124 min was used at a flow rate of 0.5 μL/min to elute peptides into the mass spectrometer. Columns were washed and re-equilibrated between LC-MS/MS experiments. Electrospray ionization was carried out at 1.7 kV using the NanoMate, with the LTQ heated capillary set to 150 °C.

Mass spectra were acquired in the orbitrap mass analyzer in the positive-ion mode over the range of *m*/*z* 300 to 2000 at a resolution of 60,000 @ *m*/*z* 400. Mass accuracy after internal calibration was within 4 ppm. Simultaneously, tandem MS spectra were acquired using the LTQ for the five most abundant, multiply charged species in the mass spectrum with signal intensities of >8000 signal/noise levels. With MS/MS collision energies set at 35%, and helium used as the collision gas, MS/MS spectra were acquired over a range of *m*/*z* values dependent on the precursor ion. Dynamic exclusion was set such that MS/MS data for each species were acquired a maximum of twice. All spectra were recorded in profile mode for further processing and analysis. Xcalibur software was used for MS and MS/MS data analysis, while peptide and protein assignments were conducted using Mascot to search against the *ScerevisiaeOrfTrans* database.

### 2.10. Mass Spectrometric Analysis of Pkc1 Phospho-Sites

Histidine-biotin tandem affinity purifications of Pkc1-HTBeaq were based on methods described elsewhere [43], with modifications below. Stable isotope labeling using amino acids in cell culture (SILAC) [47] was achieved as described previously [43]. Cells expressing Pkc1 C-terminally tagged with a HTBeaq tag [43] were grown to mid-logarithmic phase (OD_600 nm_ = 2.0), treated with 200 mM HU for 4 h, harvested by filtration, and rapidly deep frozen in liquid N_2_. In-solution digestion with trypsin and enrichment of phosphorylated peptides using TiO_2_ was performed as described previously [43]. 

For these mass spectrometry analyses, a Q Exactive HF Orbitrap (Thermo Fisher Scientific) mass spectrometer was used with the following settings: Peptides were separated applying an increasing organic solvent (acetonitrile) gradient from 2.5% to 40% in 0.1% formic acid over 60 (YPD) or 120 (SILAC) minutes at a flow rate of 275 nl/min. MS1 resolution was set to 70,000 @ *m*/*z* 200, AGC 3 × 10^6^. MS2 resolution was set to 17,500 @ *m*/*z* 200, AGC 1 × 10^5^, 500 ms max. ion injection time (IIT). The mass spectrometer was configured to pick the twelve most abundant precursor ions for data-dependent MS2 scans, applying HCD for fragmentation with a normalized collision energy (NCE) of 27. Dynamic exclusion time was set to 30 s. MS raw files were processed using MaxQuant (Max Planck Institute of Biochemistry, Munich) [48] software version 1.5.2.8 using standard settings, except for the following modifications. Spectra were searched against the *Saccharomyces* Genome Database (http://www.yeastgenome.org/ accessed on 8 October 2015) containing 6717 entries (3 February 2011), including a list of 248 common laboratory contaminants as well as reversed versions of all sequences. The enzyme specificity was set to trypsin. A maximum of two missed cleavages was allowed. Phosphorylation of serine, threonine, and tyrosine residues, oxidation of methionine, and deamidation of asparagine were set as variable modifications. For stable isotope labeling using amino acids in cell culture (SILAC)–labeled samples, Lys6 and Arg6 were additionally selected. Carbamidomethylation of cysteine was searched as a fixed modification. A maximum of five modifications per peptide was allowed. The false discovery rate for peptide, protein, and site identification was set to 1%. All files were searched together. Minimum delta score for modified peptides was set to 6. The MS phospho-proteomics data have been deposited at the zenodo repository (https://zenodo.org/ accessed on 10 September 2021) and can be accessed via https://doi.org/10.5281/zenodo.5552273.

### 2.11. Notes on Reproducibility

All immunoblots, and co-IPs, were reproduced at least once in independent experiments with representative images shown. 

## 3. Results and Discussion

**Figure 1:**
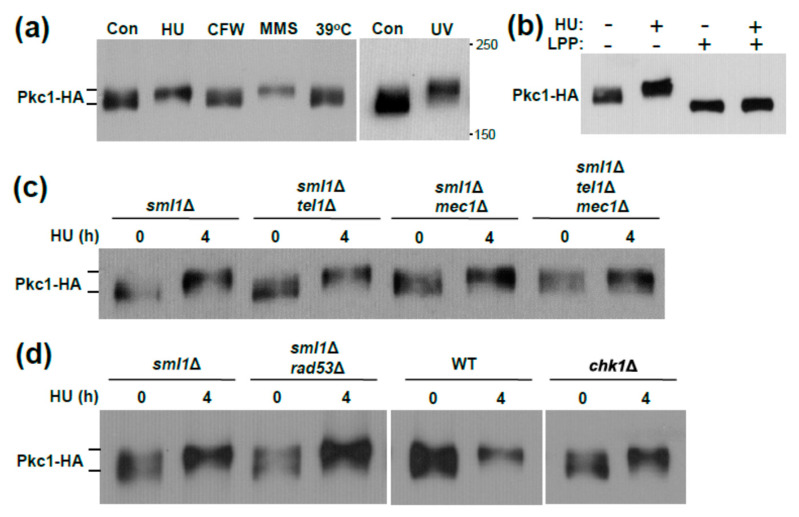
A phosphorylation band-shift in Pkc1 requires the *MEC1* and *TEL1* DNA damage checkpoint genes. (**a**) A Pkc1 band-shift is induced specifically by genotoxic stressors. Wild-type cells (DL3950; *sml1*Δ) expressing Pkc1-HA (from p813) were exposed to genotoxic stress (250 mM HU for 4 h, 0.04% MMS for 2 h, or 150 J/m^2^ UV with a 2 h recovery period), cell wall stress (40 µg/mL CFW for 1 h, or heat shock at 39 °C for 1 h), or untreated (Con). Extracts were subjected to SDS-PAGE and immunoblot analysis for Pkc1-HA; (**b**) Phosphorylation is responsible for the HU-induced band-shift. Wild-type cells (DL3950) expressing Pkc1-HA were either treated with HU as above, or untreated. Extracts were treated with Lambda protein phosphatase (LPP), as described in Methods, prior to immunoblot analysis; (**c**,**d**) The HU-induced Pkc1 band-shift requires *MEC1* and *TEL1*, but not the checkpoint genes that they regulate (*RAD53* or *CHK1*). Cultures were treated with HU as above prior to immunoblot analysis for Pkc1-HA. Strains are DL3950 (*sml1*Δ), DL3951 (*sml1*Δ *tel1*Δ), DL3952 (*sml1*Δ *mec1*Δ), DL4277 (*sml1*Δ *mec1*Δ *tel1*Δ), DL3953 (*sml1*Δ *rad53*Δ), DL2772 (Res. Gen. WT), and DL4286 (Res. Gen. *chk1*Δ). The *sml1*Δ mutation is required to suppress the lethality of the *mec1*Δ and *rad53*Δ mutations.

Pkc1 undergoes a phosphorylation-induced band-shift in response to DNA damage [30]. We found that the Pkc1 band-shift was induced specifically in response to DNA damage by alkylating agent methylmethane sulfonate (MMS), by dNTP depletion by hydroxyurea (HU) treatment, or by UV irradiation, but not in response to cell wall stress treatments calcofluor white (CFW) or elevated growth temperature (Figure 1a). We confirmed that this band-shift results from hyper-phosphorylation by treatment with Lambda protein phosphatase (Figure 1b). We also found that it is dependent on the partially redundant DNA damage checkpoint kinases Mec1 and Tel1 (Figure 1c). This is in contrast to the findings of Soriano-Carot, et al. [30], who found that Tel1 was uniquely responsible for the Pkc1 band-shift. In any case, we found that both of the known effector kinase targets of the checkpoint kinases, Rad53 and Chk1 [49,50], were dispensable for the Pkc1 band-shift (Figure 1d). These results suggest that Mec1 and Tel1 either phosphorylate Pkc1 directly, or act on another protein kinase that phosphorylates Pkc1. Thus, the DNA damage checkpoint kinases, Mec1 and Tel1, regulate hyper-phosphorylation of Pkc1 in response to genotoxic stress.

### 3.1. Genotoxic Stress Induces Hrr25 Association with Pkc1

**Figure 2:**
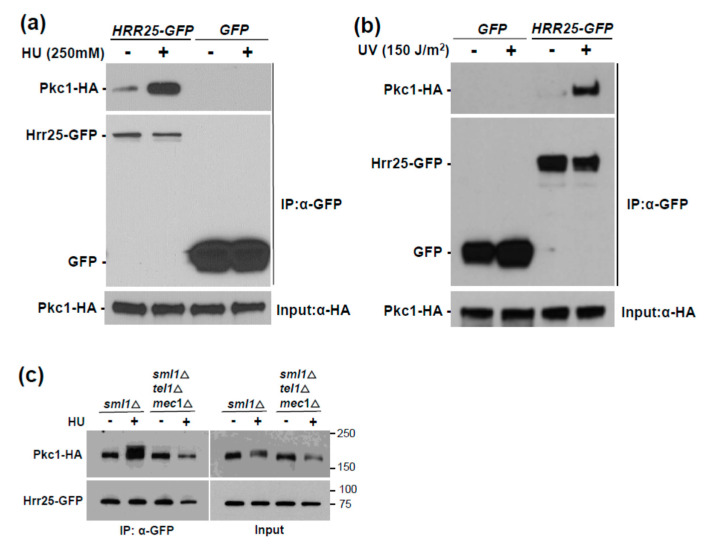
HU treatment induces association of Pkc1 with Hrr25. (**a**) Wild-type cells (DL100) co-expressing Pkc1-HA (from p813) and Hrr25-GFP (from p3357) or GFP (from p3358) were treated with 250 mM HU for 4 h. Hrr25-GFP or GFP was immunoprecipitated (IP) from extracts and samples were tested by immunoblot analysis for co-IP of Pkc1-HA. Input Pkc1-HA from extracts is shown at bottom; (**b**) UV treatment induces association of Pkc1 with Hrr25. Wild-type cells (DL100) co-expressing Pkc1-HA (from p813) and Hrr25-GFP (from p3357) or GFP (from p3358) were treated with UV light (150 J/m^2^) and returned to culture for 2 h post-irradiation prior to extract preparation. Hrr25-GFP or GFP was immunoprecipitated (IP) from extracts and treated as above; (**c**) *MEC1* and *TEL1* are required for the HU-induced association of Hrr25 with Pkc1. Cultures co-expressing Pkc1-HA and Hrr25-GFP (from p3562) were treated with HU as above and processed for co-IP of Pkc1-HA with Hrr25-GFP. Strains were DL3950 (*sml1*Δ) and DL4277 (*sml1*Δ *tel1*Δ *mec1*Δ). Molecular mass markers (in kDa) are shown on the right.

Next, we took a proteomics approach to identify protein kinases that associate with Pkc1 in response to HU-induced genotoxic stress. Cells expressing Pkc1-GFP were treated with 250 mM HU for 4 h, or were untreated, and Pkc1-GFP was immunoprecipitated from extracts and subjected to mass spectrometric (MS) analysis to identify co-precipitating proteins. Proteins that were found associated with Pkc1 only after HU treatment, or only without HU treatment are presented in Supplemental Tables S1 and S2, respectively. Although numerous non-specific associations were detected in both the untreated and treated samples, a minor signal from the DNA damage and repair kinase Hrr25 (HO and radiation repair) [51] was detected only in the HU-treated sample. *HRR25* encodes an isoform of casein kinase 1 (CK1) that has been implicated in the repair of DNA double strand breaks [51] and is required for the transcriptional induction of the ribonucleotide reductase genes *RNR2* and *RNR3* in response to DNA damage [52]. Many physical interactors have been identified for Pkc1, but Hrr25 is not among them (*Saccharomyces* Genome Database). Therefore, we validated the physical association between Pkc1 and Hrr25 by co-immunoprecipitation (co-IP). Not only did we detect a stable interaction between Pkc1-HA and Hrr25-GFP, but the interaction was reproducibly induced 5–10-fold in response to treatment with HU (Figure 2a). We detected no non-specific binding of Pkc1-HA to either GFP alone (Figure 2a), or to the GFP-trap beads (Supplemental Figure S1). Hrr25 was recruited similarly to Pkc1 in response to ultraviolet (UV) light exposure (Figure 2b), suggesting that this is part of the generalized response to genotoxic stress.

**Figure 3:**
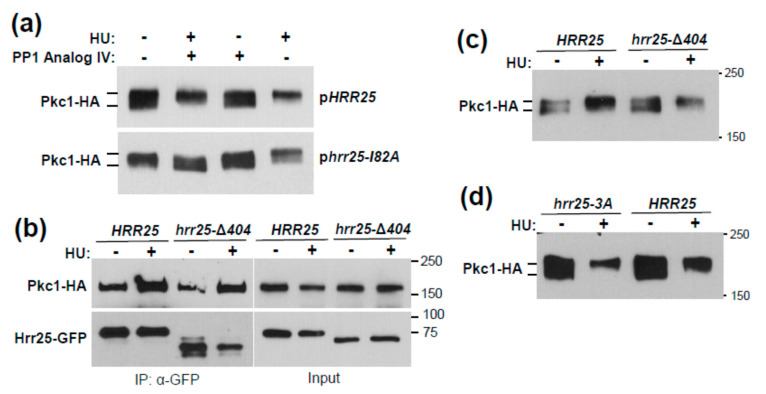
An analog-sensitive form of Hrr25 shows that its catalytic activity is required for the HU-induced Pkc1 band-shift. (**a**) An *hrr25*Δ strain complemented by plasmid-borne *HRR25* (DL4527) or *hrr25-182A* (DL4528; encoding an analog-sensitive form) and expressing Pkc1-HA (from p813) was treated simultaneously with HU (250 mM) and/or PP1 analog IV (20 μM) for 4 h. Extracts were processed for immunoblot analysis of Pkc1-HA; (**b**) A C-terminal truncation of Hrr25 lacking three potential Mec1/Tel1 phosphorylation sites associates normally with Pkc1. An *hrr25*Δ strain complemented by plasmid-borne *HRR25-GFP* (DL4541) or *hrr25-*Δ*404* (DL4542) and expressing Pkc1-HA (from p813) was treated with HU (250 mM for 4 h). Hrr25-GFP was immunoprecipitated from extracts and tested for co-IP of Pkc1-HA by immunoblot analysis. Molecular mass markers (in kDa) are on the right; (**c**) A C-terminal truncation of Hrr25 does not impact the HU-induced Pkc1 band-shift. An *hrr25*Δ strain complemented by plasmid-borne *HRR25* (DL4527) or *hrr25-*Δ*404* (DL4555) and expressing Pkc1-HA was treated with HU as above and processed for immunoblot analysis of Pkc1-HA (**d**) A mutant form of *HRR25* lacking three potential Mec1/Tel1 phosphorylation sites does not impact the HU-induced Pkc1 band-shift. An *hrr25*Δ strain complemented by plasmid-borne *HRR25* (DL4527) or *hrr25-3A* (DL4556) and expressing Pkc1-HA was treated with HU as above and processed for immunoblot analysis of Pkc1-HA.

We next tested the role of Mec1 and Tel1 in the induced association of Hrr25 with Pkc1. Here, we found that, not only are the DNA damage checkpoint kinases required for the Pkc1 phosphorylation band-shift, but they are also required for the induced association of Hrr25 with Pkc1 (Figure 2c), suggesting that Mec1 and Tel1 induce the Pkc1 phosphorylation band-shift indirectly by driving its association with Hrr25. Therefore, we sought to test this idea using mutants in *HRR25*. Because the *HRR25* gene is essential for viability, we constructed two analog-sensitive “gatekeeper” alleles [53] of *HRR25* (*hrr25-I82A* and *hrr25-I82G*). These were introduced on plasmids into an *hrr25*Δ strain that is maintained with a galactose-inducible form of *HRR25* and tested for their sensitivity to a collection of protein kinase inhibitory ATP-analogs in glucose-containing medium (YPD). We determined that the *hrr25-I82A* allele conferred optimum growth sensitivity to PP1 analog IV, which did not appreciably inhibit the growth of the strain expressing wild-type *HRR25* (Supplemental Figure S2). We next introduced the *hrr25-I82A* allele to an *hrr25*Δ strain by plasmid shuffle (See Materials and Methods, Section 2) to test the importance of this protein kinase in the HU-induced Pkc1 band-shift. The strain expressing the *hrr25-I82A* allele along with an epitope tagged form of Pkc1, was subjected to inhibition of Hrr25, together with HU treatment to induce genotoxic stress. Pkc1 failed to display an HU-induced band-shift in the *hrr25-I82A* strain in the presence of inhibitor, in contrast to the *HRR25* strain (Figure 3a). This suggests that Hrr25 catalytic activity is, indeed, required for the Pkc1-bandshift observed in response to genotoxic stress.

Hrr25 possesses three potential Mec1/Tel1 sites (S/T-Q) [21], which all reside within the C-terminal tail of Hrr25 (residues S405, S438, and T453). Therefore, we generated a truncated version of Hrr25 that is missing the C-terminal domain from residues 405–494 (*hrr25-*Δ*404*), which removes all three potential Mec1/Tel1 sites. This allele complemented the lethality of the *hrr25*Δ mutation, therefore we tested it for HU-induced association with Pkc1 and for the HU-induced Pkc1 band-shift. The truncated form of Hrr25 was recruited normally to Pkc1 in response to HU treatment (Figure 3b) and retained its ability to drive the Pkc1 band-shift (Figure 3c). However, we considered the possibility that the Hrr25 C-terminal region might carry an auto-inhibitory domain, truncation of which could activate Hrr25 independently of Mec1/Tel1. To address this possibility, we mutated the three potential Mec1/Tel1 sites within this domain to Ala residues, yielding *hrr25-3A*, and tested the influence of this allele on the HU-induced Pkc1 band-shift. As with the truncated allele, the *hrr25-3A* mutant was able to mediate the HU-induced band-shift normally (Figure 3d). Therefore, we conclude that Mec1 and Tel1 likely regulate HU-induced Hrr25 association with Pkc1 and the Pkc1 band-shift by means other than direct phosphorylation of Hrr25. Because neither Rad53, nor Chk1, the known effector kinases of Mec1 and Tel1, were required for the HU-induced Pkc1 band-shift, it remains unclear how the sensor kinases regulate the Hrr25 action on Pkc1. It is possible that Mec1 and Tel1 act on Pkc1, rather than on Hrr25, to regulate the association of these protein kinases. Nevertheless, our results strongly suggest that both Hrr25 and Pkc1 are indirect Mec1/Tel1 effectors.

### 3.2. Identification of Pkc1 Phospho-Sites in Response to HU Treatment

Hrr25 is an ortholog of mammalian casein kinase 1 delta (CK1δ) [54]. Two phosphorylation site motifs for this class of protein kinase have been described [55,56,57,58]. These protein kinases require either a priming phosphorylation at position −3 relative to the target S/T site (i.e., p-S/TXXS/T) or an acidic residue at position −3 relative to the target site (i.e., D/EXXS/T). 

**Figure 4:**
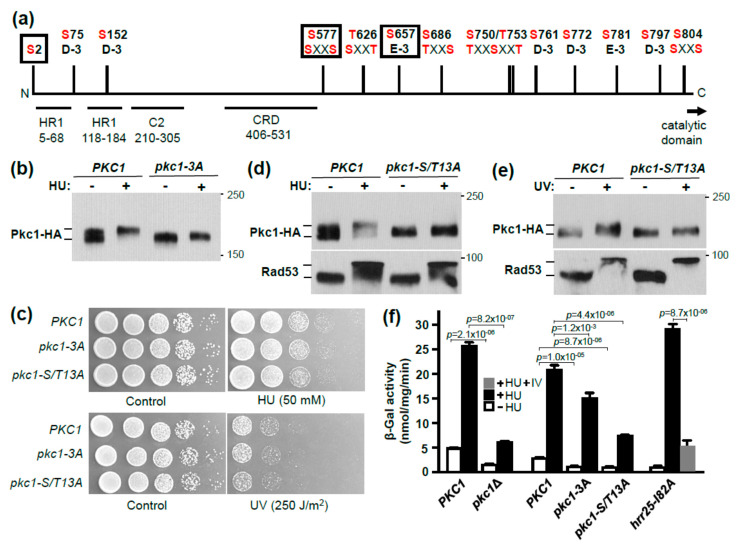
CK1 phosphorylation sites within the Pkc1 regulatory domain are responsible for the genotoxic stress-induced Pkc1 band-shift. (**a**) Phosphorylation sites within the Pkc1 regulatory domain mutated in this study. Phosphorylated residues are marked in red. The three residues mutated in the *pkc1-3A* allele (S2, S577, and S657) are marked by boxes and were identified by SILAC MS as increased in phosphorylation state in response to HU treatment. Two of these residues (S577 and S657) are within consensus CK1 phosphorylation sites, with either a priming phospho-Ser at position −3 (S577) or an acidic residue (Asp) at position −3 (S657). Other phosphorylated residues that reside within CK1 consensus sites are also indicated and were mutated in the *pkc1-S/T13A* allele. Known regulatory elements, Rho-binding domains (HR1), calcium/lipid-binding domain (C2), and Cys-rich domain (CRD) are also shown. The catalytic domain is C-terminal to the regulatory domain and starts at residue 824; (**b**) HU-induced phosphorylation band-shift of the Pkc1-3A mutant. Plasmids were *PKC1-HA* (p813) and *pkc1-3A-HA* (p3619) (**c**) The *pkc1-3A* and *pkc1-S/T13A* mutants do not show increased sensitivity to genotoxic stress. Serial 10-fold dilutions of cultures grown to mid-log phase in YPD were spotted onto plates (left to right) with or without HU. Cultures treated with UV were similarly diluted and spotted onto YPD plates. Plates were incubated at 25 °C for two days. Plasmids were *PKC1-HA* (p3623), *pkc1-3A* (p3624), and *pkc1-S/T13A* (p3625); (**d**) HU-induced phosphorylation band-shift of the Pkc1-S/T13A mutant and Rad53. Plasmids were *PKC1-HA* (p813) *pkc1-S/T13A* (p3612); (**e**) UV-induced phosphorylation band-shift of the Pkc1-S/T13A mutant and Rad53. Plasmids were *PKC1-HA* (p813) and *pkc1-S/T13A* (p3612). Strain DL1021 (*pkc1*Δ) was used for experiments shown in (**b**,**d**,**e**). Strain DL376 (*pkc1*Δ) was used for the experiment in panel (**c**); (**f**) HU-induced *RNR3-lacZ* expression is diminished in a *pkc1*Δ mutant, and in the *pkc1-3A* and *pkc1-S/T13A* mutants. Strain DL376 (*pkc1*Δ) was co-transformed with p*RNR3-lacZ* (p2947) and *PKC1-HA* (p3623), *pkc1-3A* (p3624), *pkc1-S/T13A* (p3625), or vector alone (p118). Cells were cultured in the presence of 0.5 M sorbitol for osmotic support (pair on left), or in the absence of sorbitol. Cultures were treated for 4 h with 250 mM HU and β-galactosidase activity was measured from extracts. The *hrr25-I82A* mutant (DL4528; right) was treated with HU plus or minus PP1 analog IV (20 μM) for 4 h. Each value is the mean and standard deviation from three independent cultures. Pair-wise *p*-values for HU-treated and untreated samples were calculated using student t-test and were all at least *p* ≤ 0.00001, except the HU-treated *PKC1* and *pkc1-3A* pair, which was *p* = 0.0012. An additional p-value of *p* < 0.00001 was obtained for the *hrr25-I82A* mutant for HU-treated samples, with and without analog IV.

We conducted both a shotgun mass spectrometric (MS) analysis and a quantitative SILAC MS analysis of HU-induced phosphorylation sites on Pkc1 [43]. These analyses identified many phosphorylation sites on Pkc1, several of which had not been described previously (i.e., T570, T626, S666, T779, S781, and T785; Supplemental Figure S3). Among the phosphorylation sites identified in our SILAC MS experiments, only three appeared to be upregulated in response to HU treatment: S2, S577, and S657 (Supplemental Table S3). Of these, both S577 and S657 are potential Hrr25 phosphorylation sites because S577 has a priming phosphorylation site (p-S574) and S657 is positioned three residues beyond E654 (Figure 4a). Therefore, we started our mutational analysis with these three sites. The individual mutations did not display a detectable impact on the HU-induced Pkc1 band-shift (not shown), so we created a triple mutant. The *pkc1-3A* (*pkc1-S2A, S577A, S657A*) allele was able to complement the lethality of a *pkc1*Δ mutant for growth on rich medium in the absence of osmotic support. Therefore, we examined its behavior in response to genotoxic stress. The HU-induced Pkc1 band-shift was somewhat diminished in the *pkc1-3A* mutant (Figure 4b), revealing a cumulative effect of these phosphorylation sites. We next tested this mutant for its sensitivity to HU and UV treatment. However, this mutant did not display increased sensitivity to either treatment (Figure 4c). Therefore, we considered a larger collection of Pkc1 phosphorylation sites. 

Among the phosphorylation sites identified in our analyses, together with those established previously [21,59,60,61], we identified 13 phosphorylation sites within Pkc1 that fit either of the two CK1 consensus sequences. Figure 4a shows a map of these sites, all of which reside within the N-terminal regulatory domain of Pkc1. However, most of these sites (11) are clustered in an area without recognizable regulatory features between residues 577 and 804. We mutated all of these phosphorylation sites to alanine residues in groups and in series. We were only able to detect an impact on the HU-induced Pkc1 band-shift once at least nine of these CK1 residues were mutated (data not shown). Blocking the HU-induced band-shift completely required mutation of all 13 sites in the *pkc1-S/T13A* mutant (Figure 4d). The UV-induced band-shift was also blocked in the *pkc1-S/T13A* mutant (Figure 4e). The DNA damage checkpoint was activated normally in the *pkc1-S/T13A* mutant, as judged by a strong Rad53 band-shift (Figure 4d,e). The *pkc1-S/T13A* allele also complemented the null mutant for growth in the absence of osmotic support. Therefore, we tested the sensitivity of this strain to genotoxic stress. However, like the *pkc1-3A* mutant, the *pkc1-S/T13A* mutant did not display enhanced sensitivity to either HU or UV treatment (Figure 4c), suggesting that the biological impact of Pkc1 hyper-phosphorylation during genotoxic stress is too subtle to detect by this viability assay. 

**Figure 5:**
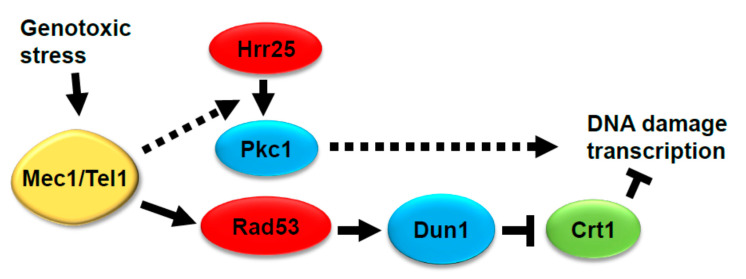
Proposed contribution of Hrr25 and Pkc1 to DNA damage-induced transcription. Pathway from Mec1 and Tel1 through Hrr25 and Pkc1 is added to the pathway established by Huang et al. [26]. Dashed arrows suggest indirect regulation.

*HRR25* has been implicated in the transcriptional response to DNA damage, most notably in the induction of the *RNR3* gene [52], but its role has not been clearly established. The cell cycle transcriptional regulatory factor SBF, comprised of Swi4 and Swi6, is similarly important for this transcriptional response and Hrr25 associates with and phosphorylates Swi6 in vitro [52]. However, *RNR3* induction in response to DNA damage was also shown to be largely under the control of the Mec1-Rad53-Dun1 pathway through the Crt1 transcriptional repressor, which is hyper-phosphorylated in response to genotoxic stress [26]. Therefore, we asked if the expression of an *RNR3-lacZ* reporter was influenced by *PKC1*. We found that both basal *RNR3-lacZ* expression and its induction by HU treatment were strongly diminished in a *pkc1*Δ mutant grown in the presence of osmotic support (Figure 4f). We also examined the effect of the *pkc1-3A* and *pkc1-S/T13A* alleles. *RNR3-lacZ* basal expression and induction were only modestly diminished in the *pkc1-3A* mutant, but were more strongly impaired in the *pkc1-S/T13A* mutant (Figure 4f). Although the basal and induced levels of *RNR3-lacZ* expression were reduced in each of these mutants, the relative induction was retained for all (approximately 6 to 8-fold). Finally, we tested the analog-sensitive *hrr25-I82A* mutant for induction of *RNR3-lacZ* expression and found that, as anticipated, inhibitor treatment strongly diminished HU induction (Figure 4f). These results suggest that Hrr25 may regulate the transcriptional response to DNA damage in part through phosphorylation of Pkc1 (Figure 5). This pathway would be independent of the Mec1-Rad53-Dun1 pathway, because Pkc1 hyper-phosphorylation in response to genotoxic stress does not require Rad53.

## 4. Conclusions

We can draw several conclusions from this study. First, the checkpoint kinases Mec1 and Tel1 regulate hyper-phosphorylation of Pkc1 under conditions of genotoxic stress by inducing the association of CK1 homolog Hrr25 with Pkc1. This happens through a mechanism that does not require the phosphorylation of Hrr25 by Mec1 or Tel1 and suggests that Hrr25 and Pkc1 are indirect effectors of the checkpoint kinases. Second, a large collection of CK1 phosphorylation sites contribute to the genotoxic stress-induced Pkc1 band-shift. Finally, CK1 phosphorylation site mutants in Pkc1 are partially deficient in *RNR3-lacZ* basal and DNA damage-induced expression, suggesting that Pkc1 hyper-phosphorylation by Hrr25 contributes to this response.

## Figures and Tables

**Table 1:** Yeast strains used in this study.

Strain	Relevant Genotype	Source or Reference
DL100	*MAT*a EG123 *ura3-52 leu2-3,112 trp1-1 his4 can1^r^*	[36]
DL376	*MAT*a EG123 *ura3-52 leu2-3,112 trp1-1 his4 pkc1*∆*::LEU2*	David Levin
DL1021	*MAT*a SEY6210 *leu2-3,112 ura3-52 his3-200 trp1-901 ede2-101 pkc1∆::HIS3 suc2-9* (GPY1115)	Gerhard Paravicini
DL2772	*MATα* S288c (BY4742) *his3 leu2 lys2 ura3*	Research Genetics
DL3950	*MATα* MBS62 *sml1*∆*::TRP1*	Marcus Smolka
DL3951	*MATα* MBS103 *sml1*∆*::TRP1 tel1*∆::*URA3*	Marcus Smolka
DL3952	*MATα* MBS104 *sml1*∆*::TRP1 mec1*∆::*KanMX*	Marcus Smolka
DL3953	*MATα* MBS72 *sml1*∆*::TRP1 rad53*∆::*HIS3*	Marcus Smolka
DL3954	*MAT*a/*α MBS115 SML1/sml1*∆*::TRP1 MEC1/mec1*∆*::HIS3 TEL1/tel1*∆*::URA3*	Marcus Smolka
DL4206	*MAT*a W303 *ade2 trp1 leu2 his3 ura3 can1*	Juan Carlos Igual
DL4277	*MATα* MBS *sml1*∆*::TRP1 mec1*∆*::HIS3 tel1*∆*::URA3*	This study
DL4286	*MATα* BY4742 *chk1*∆*::KanMX*	Research Genetics
DL4290	*MAT*a *ura3-52 lys2-801 ade2-101 trp1-*∆*63 his3-*∆*200 leu2-*∆*1 hrr25*∆*::loxP-kanMX-loxP pGAL1-3HA-HRR25^degron^* (KKY387)	[37]
DL4503	*MATα* MBS *sml1*∆*::TRP1 mec1*∆::*KanMX tel1*∆::*URA3*	This study
DL4515	*MAT*a W303 *hrr25*∆::*HPHMX4* (p*HRR25-HA*; p3484, *LEU2 2µ*)	This study
DL4527	*MAT*a W303 *hrr25*∆::*HPHMX4* (p*HRR25-HA*; p3545, *HIS3 CEN*)	This study
DL4528	*MAT*a W303 *hrr25*∆::*HPHMX4* (p*hrr25*-*I82A*-*HA*; p3550, *HIS3 CEN*)	This study
DL4541	*MAT*a W303 *hrr25*∆::*HPHMX4* (p*HRR25-GFP*; p3562, *HIS3 2µ*)	This study
DL4542	*MAT*a W303 *hrr25*∆::*HPHMX4* (p*hrr25-∆404*-*GFP*; p3567, *HIS3 2µ*)	This study
DL4555	*MAT*a W303 *hrr25*∆::*HPHMX4* (p*hrr25*-*∆404-HA*; p3546, *HIS3 CEN*)	This study
DL4556	*MAT*a W303 *hrr25*∆::*HPHMX4* (p*hrr25-3A*-*HA*; p3576, *HIS3 CEN*)	This study
JV826	*MAT*a BY4741 *PKC1-HTBeaq::NatMX*	This study

**Table 2:** Plasmids used in this study.

Plasmid	Description	Source or Reference
p117	pRS313	[39]
p118	pRS314	[39]
p119	pRS315	[39]
p120	YEp351	[40]
p813	YEp351-*PKC1-HA*	David Levin
p1105	pRS425	[39]
p1202	pRS425-*GFP*	David Levin
p2062	pVDG7 *PKC1-GFP*	[41]
p2454	pRS413	[39]
p2947	p*RNR3-lacZ*	Stephen Elledge
p3064	pAG32-*RGC2*	[42]
p3149	pRS425-*3HA-ADH1^T^*	[42]
p3357	pUG36-*HRR25-GFP*	Martha Cyert
p3358	pUG36-*GFP*	Martha Cyert
p3484	pRS425-*HRR25-HA*	This study
p3504	pRS313-*3HA-ADH1^T^*	This study
p3517	YEp351-*pkc1-S577A*-*HA*	This study
p3521	YEp351-*pkc1-S577A*, *S626A*-*HA*	This study
p3522	YEp351-*pkc1-S577A*, *T626A*, *T753A-HA*	This study
p3523	YEp351-*pkc1-S577A*, *T626A*, *T753A*, *S804A*-*HA*	This study
p3525	pRS315-*HRR25-HA*	This study
p3538	pRS425-*hrr25-*∆*404*-*HA*	This study
p3544	pRS423-*3HA-ADH1^T^*	This study
p3545	pRS313-*HRR25-HA*	This study
p3546	pRS313-*hrr25-*∆*404*-*HA*	This study
p3547	pRS423-*HRR25-HA*	This study
p3550	pRS313-*hrr25-I82A*-*HA*	This study
p3552	pRS423-*hrr25-I82A*-*HA*	This study
p3553	pRS423-*hrr25-I82G*-*HA*	This study
p3560	pRS423-*GFP*	This study
p3562	pRS423-*HRR25-GFP*	This study
p3567	pRS423-*hrr25-*∆*404*-*GFP*	This study
p3570	pRS313-*hrr25-T453A*-*HA*	This study
p3572	pRS313-*hrr25*-*T453A*, *S405A*-*HA*	This study
p3574	YEp351-*pkc1-S577A*, *T626A*, *T753A*, *S761A*, *S804A*-*HA*	This study
p3576	pRS313-*hrr25*-*T453A*, *S405A*, *S438A*-*HA*	This study
p3597	YEp351-*pkc1-S577A*, *T626A*, *T753A*, *S761A*, *S772A*, *S804A*-*HA*	This study
p3603	YEp351-*pkc1-S577A*, *T626A*, *S686A*, *T753A*, *S761A*, *S772A*, *S804A*-*HA*	This study
p3604	YEp351-*pkc1-S577A*, *T626A*, *S686A*, *S750A*, *T753A*, *S761A*, *S772A*, *S804A*-*HA*	This study
p3605	YEp351-*pkc1-S152A*, *S577A*, *T626A*, *S686A*, *S750A*, *T753A*, *S761A*, *S772A*, *S804A*-*HA*	This study
p3606	YEp351-*pkc1-S152A*, *S577A*, *T626A*, *S657A*, *S686A*, *S750A*, *T753A*, *S761A*, *S772A*, *S804A*-*HA*	This study
p3608	YEp351-*pkc1-S152A*, *S577A*, *T626A*, *S657A*, *S686A*, *S750A*, *T753A*, *S761A*, *S772A*, *S781A*, *S804A*-*HA*	This study
p3610	YEp351-*pkc1-S75A*, *S152A*, *S577A*, *T626A*, *S657A*, *S686A*, *S750A*, *T753A*, *S761A*, *S772A*, *S781A*, *S804A*-*HA*	This study
p3612	YEp351-*pkc1-S75A*, *S152A*, *S577A*, *T626A*, *S657A*, *S686A*, *S750A*, *T753A*, *S761A*, *S772A*, *S781A*, *S797A*, *S804A*-*HA* (S/T13A)	This study
p3617	YEp351-*pkc1-S2A-HA*	This study
p3618	YEp351-*pkc1-S2A*, *S657A-HA*	This study
p3619	YEp351-*pkc1-S2A*, *S657A*, *S577A-HA* (S3A)	This study
p3623	pRS314-*PKC1-HA*	This study
p3624	pRS314-*pkc1*-*S2A*, *S657A*, *S577A-HA* (S3A)	This study
p3625	pRS314-*pkc1-S75A*, *S152A*, *S577A*, *T626A*, *S657A*, *S686A*, *S750A*, *T753A*, *S761A*, *S772A*, *S781A*, *S797A*, *S804A*-*HA* (S/T13A)	This study
pWR268	pFA6a-integrative *HTBeaq* tag, *NatMX*	[43]

## Data Availability

The MS phospho-proteomics data have been deposited at the zenodo repository (https://zenodo.org/ accessed on 10 September 2021) and can be accessed via 10.5281/zenodo.5102666.

## Supplementary Materials

The following are available online at https://www.mdpi.com/article/10.3390/jof7100874/s1, Table S1: Pkc1 MS with HU only, Table S2: Pkc1 MS without HU only, Table S3: SILAC MS Pkc1 with and without HU, Figure S1: Binding of Hrr25 to Pkc1. Figure S2: Sensitivity of *hrr25* “gatekeeper” mutants to growth inhibition by inhibitory ATP analogs, Figure S3: Pkc1 phosphorylation sites identified in this study.
