# Supplementary material for: Regulation of Pkc1 Hyper-Phosphorylation by Genotoxic Stress
Source: J Fungi (Basel). 2021 Oct 17;7(10):874. doi: 10.3390/jof7100874 (PMC8541566; doi:10.3390/jof7100874)
Supplement: Supplementary file 1 [file jof-07-00874-s001.zip › jof-1397680-supplementary.pdf]

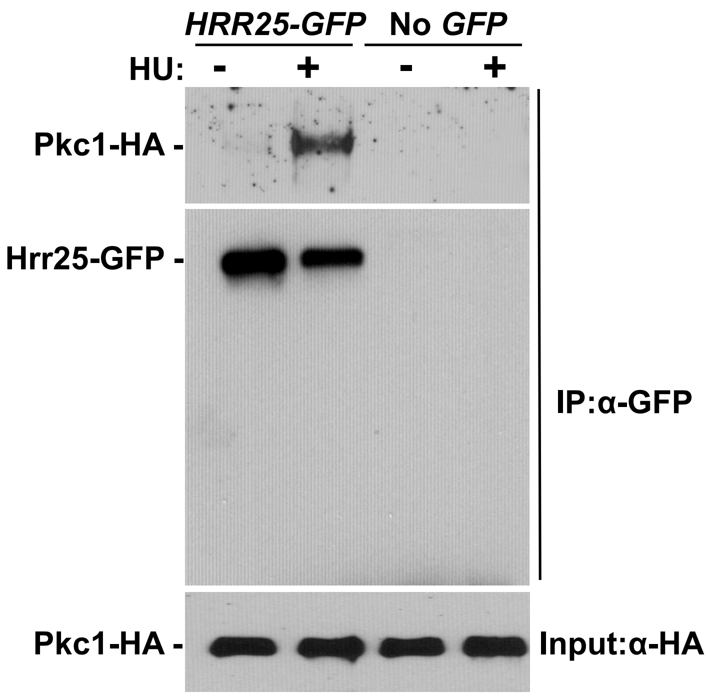

Supplemental Figure S1. HU treatment induces association of Pkc1 with Hrr25. Wild-type cells (DL100) co-expressing Pkc1-HA (from p813) and Hrr25-GFP (from p3357), or Pkc1-HA alone, were treated with 250 mM HU for 4 h. Hrr25-GFP was immunoprecipitated (IP) from extracts with GFP-trap beads and samples were tested by immunoblot analysis for co-IP of Pkc1-HA. Input Pkc1-HA from extracts is shown at bottom. Non-specific binding of Pkc1-HA to beads was not detected.

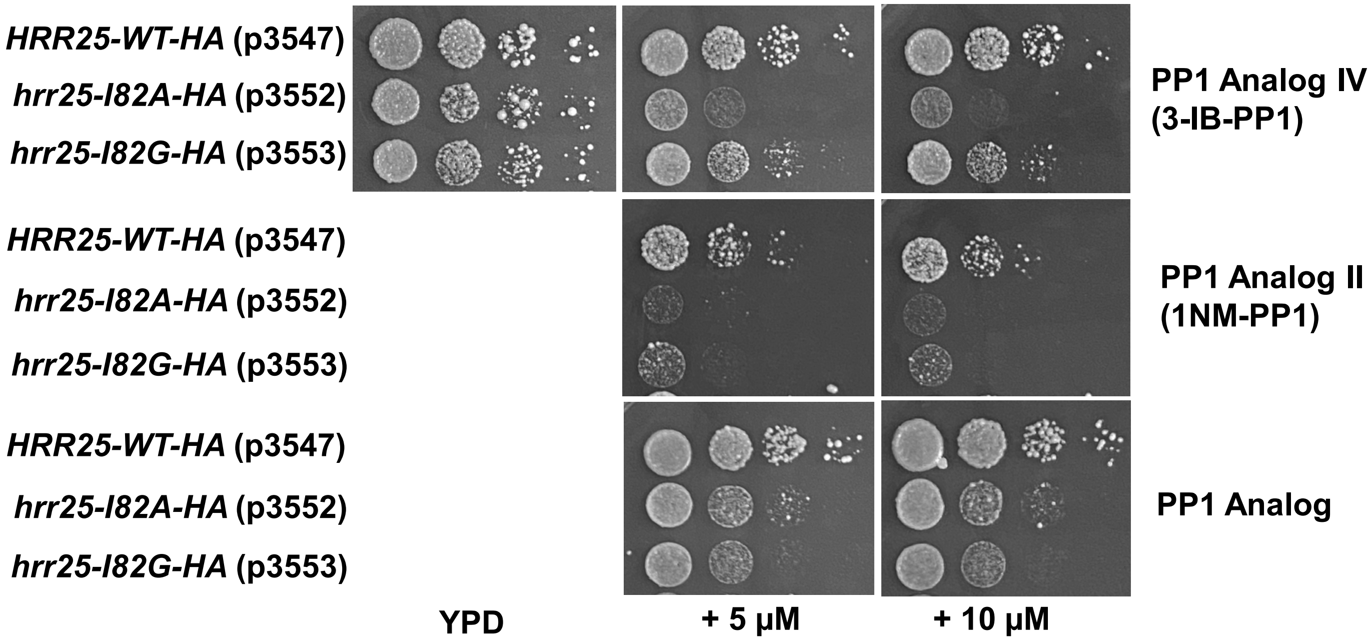

Supplemental Figure S2. Sensitivity of *hrr25* "gatekeeper" mutants to growth inhibition by inhibitory ATP analogs. Plasmids with the indicated alleles of *HRR25* were introduced into strain DL4290, which bears a chromosomal *hrr25*Δ mutation maintained by a plasmid-borne copy of $HRR25^{degron}$ under the inducible control of the *GAL1* promoter. This strain is only viable on galactose-containing medium. Transformants were plated at 10-fold dilutions (from left to right) on YPD, or YPD plus the indicated concentration of one of three protein kinase inhibitors and incubated for 3 days at 30 ^{o}C.

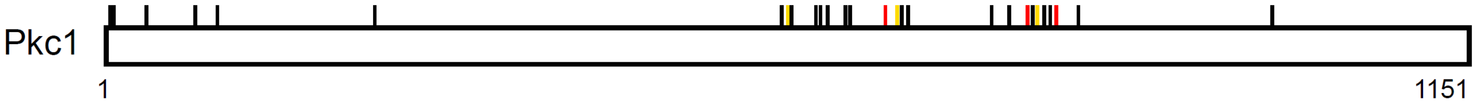

1 M**S**F**S**QLEQNI KKKIAVEENI IRGASALKKK T**S**NVMVIQKC NTNIREARQN LEYLEDSLKK

61 LRLKTAQQSQ GENG**S**EDNER CNSKEYGFLS TK**S**PNEHIFS RLDLVKYDCP SLAQRIQYML

121 QQLEFKLQVE KQYQEANTKL TKLYQIDGDQ RSSSAAEGGA MESKYRIQML NKALKKYQAI

181 NVDFDQFKHQ PNDIMDNQQP KFRRKQLTGV LTIGITAARD VDHIQ**S**PMFA RKPESYVTIK

241 IDDTIKARTK PSRNDRWSED FQIPVEKGNE IEITVYDKVN DSLIPVAIMW LLLSDIAEEI

301 RKKKAGQTNE QQGWVNASNI NGGSSLASEE GSTLTSTYSN SAIQSTSAKN VQGENTSTSQ

361 ISTNSWFVLE PSGQILLTLG FHKSSQIERK QLMGGLHRHG AIINRKEEIF EQHGHHFVQK

421 SFYNIMCCAY CGDFLRYTGF QCQDCKFLCH KKCYTNVVTK CIAKTSTDTD PDEAKLNHRI

481 PHRFLPTSNR GTKWCCHCGY ILPWGRHKVR KCSECGIMCH AQCAHLVPDF CGMSMEMANK

541 ILKTIQDTKR NQEKKKRTVP SAQLGSSIG**T** ANG**S**DL**S**PSK LAERANAPLP PQPRKHDK**T**P

601 **S**PQKVGRD**S**P TKQHDPIIDK RI**S**LQ**T**HGRE KLNKFIDENE AYLNFTEGAQ QTAEFS**S**PEK

661 TLDPT**S**NRR**S** LGLTDL**S**IEH SQTWESKDDL MRDELELWKA QREEMELEIK QDSGEIQEDL

721 EVDHIDLETK QKLDWENKND FREADL**T**IDS THTNPFRDMN **S**ETFQIEQDH ASKEVLQE**T**V

781 **S**LAP**T**STHA**S** RTTDQQ**S**PQK **S**QTSTSAKHK KRAAKRRKV**S** LDNFVLLKVL GKGNFGKVIL

841 SKSKNTDRLC AIKVLKKDNI IQNHDIESAR AEKKVFLLAT KTKHPFLTNL YCSFQTENRI

901 YFAMEFIGGG DLMWHVQNQR LSVRRAKFYA AEVLLALKYF HDNGVIYRDL KLENILLTPE

961 GHIKIADYGL CKDEMWYGNR TS**T**FCGTPEF MAPEILKEQE YTKAVDWWAF GVLLYQMLLC

1021 QSPFSGDDED EVFNAILTDE PLYPIDMAGE IVQIFQGLLT KDPEKRLGAG PRDADEVMEE

1081 PFFRNINFDD ILNLRVKPPY IPEIKSPEDT SYFEQEFTSA PPTLTPLPSV LTTSQQEEFR

1141 GFSFMPDDLD L*

Supplemental Figure S3. Pkc1 phosphorylation sites identified in this study. Ser and Thr residues that are shown in bold indicate identified phosphorylation sites according to the following confidence levels: black: >95% site confidence; red: >85% site confidence; yellow: >75% site confidence. Red asterisks indicate sites that have only been found in experiments in which cells were grown in YPD (rather than in SILAC experiments). Black lines indicate sequence coverage.

Supplemental Table S1. Pkc1-associated proteins plus HU only

Description
TDH2 SGDID:S000003769, Chr X from 454681-453683, Genome Release 64-1-1, reverse complement, Verified ORF, ""Glyceraldehyde-3-phosphate dehydrogenase, isozyme 2, involved in glycolysis and gluconeogenesis; tetramer that catalyzes the reaction of glyce
TDH1 SGDID:S000003588, Chr X from 338271-339269, Genome Release 64-1-1, Verified ORF, ""Glyceraldehyde-3-phosphate dehydrogenase, isozyme 1, involved in glycolysis and gluconeogenesis; tetramer that catalyzes the reaction of glyceraldehyde-3-phosphat
ACT1 SGDID:S000001855, Chr VI from 54377-53260,54696-54687, Genome Release 64-1-1, reverse complement, Verified ORF, ""Actin, structural protein involved in cell polarization, endocytosis, and other cytoskeletal functions""
RPS0B SGDID:S000004038, Chr XII from 242232-242321,242681-243349, Genome Release 64-1-1, Verified ORF, ""Protein component of the small (40S) ribosomal subunit, nearly identical to Rps0Ap; required for maturation of 18S rRNA along with Rps0Ap; deleti
RPL27A SGDID:S000001052, Chr VIII from 126521-126551,127113-127492, Genome Release 64-1-1, Verified ORF, ""Protein component of the large (60S) ribosomal subunit, nearly identical to Rpl27Bp and has similarity to rat L27 ribosomal protein""
YNL054W-B SGDID:S000007385, Chr XIV from 519456-520760,520762-524706, Genome Release 64-1-1, pseudogene, ""TyB Gag-Pol protein; proteolytically processed to make the Gag, RT, PR, and IN proteins that are required for retrotransposition""
RPS15 SGDID:S000005400, Chr XV from 253577-253149, Genome Release 64-1-1, reverse complement, Verified ORF, ""Protein component of the small (40S) ribosomal subunit; has similarity to E. coli S19 and rat S15 ribosomal proteins""
RNR3 SGDID:S000001328, Chr IX from 240708-238099, Genome Release 64-1-1, reverse complement, Verified ORF, ""Minor isoform of the large subunit of ribonucleotide-diphosphate reductase; the RNR complex catalyzes rate-limiting step in dNTP synthesis, r
YHB1 SGDID:S000003466, Chr VII from 959904-961103, Genome Release 64-1-1, Verified ORF, ""Nitric oxide oxidoreductase, flavohemoglobin involved in nitric oxide detoxification; plays a role in the oxidative and nitrosative stress responses""
GFA1 SGDID:S000001587, Chr XI from 245373-243220, Genome Release 64-1-1, reverse complement, Verified ORF, ""Glutamine-fructose-6-phosphate amidotransferase, catalyzes the formation of glucosamine-6-P and glutamate from fructose-6-P and glutamine in
RPL31A SGDID:S000002233, Chr IV from 322226-322282,322704-322988, Genome Release 64-1-1, Verified ORF, ""Protein component of the large (60S) ribosomal subunit, nearly identical to Rpl31Bp and has similarity to rat L31 ribosomal protein; associates w
SAR1 SGDID:S000006139, Chr XVI from 138698-138725,138865-139409, Genome Release 64-1-1, Verified ORF, ""GTPase, GTP-binding protein of the ARF family, component of COPII coat of vesicles; required for transport vesicle formation during ER to Golgi pr
ERB1 SGDID:S000004652, Chr XIII from 370517-368094, Genome Release 64-1-1, reverse complement, Verified ORF, ""Constituent of 66S pre-ribosomal particles, forms a complex with Nop7p and Ytm1p that is required for maturation of the large ribosomal sub
RET1 SGDID:S000005733, Chr XV from 733457-730008, Genome Release 64-1-1, reverse complement, Verified ORF, ""Second-largest subunit of RNA polymerase III, which is responsible for the transcription of tRNA and 5S RNA genes, and other low molecular we
KAR2 SGDID:S000003571, Chr X from 381327-383375, Genome Release 64-1-1, Verified ORF, ""ATPase involved in protein import into the ER, also acts as a chaperone to mediate protein folding in the ER and may play a role in ER export of soluble proteins;
PGK1 SGDID:S000000605, Chr III from 137746-138996, Genome Release 64-1-1, Verified ORF, ""3-phosphoglycerate kinase, catalyzes transfer of high-energy phosphoryl groups from the acyl phosphate of 1,3-bisphosphoglycerate to ADP to produce ATP; key enz
RPL40B SGDID:S000001802, Chr XI from 618374-617996,618750-618743, Genome Release 64-1-1, reverse complement, Verified ORF, ""Fusion protein, identical to Rpl40Ap, that is cleaved to yield ubiquitin and a ribosomal protein of the large (60S) ribosomal
FBA1 SGDID:S000001543, Chr XI from 327487-326408, Genome Release 64-1-1, reverse complement, Verified ORF, ""Fructose 1,6-bisphosphate aldolase, required for glycolysis and gluconeogenesis; catalyzes conversion of fructose 1,6 bisphosphate to glycera
TUB1 SGDID:S000004550, Chr XIII from 99259-97941,99400-99376, Genome Release 64-1-1, reverse complement, Verified ORF, ""Alpha-tubulin; associates with beta-tubulin (Tub2p) to form tubulin dimer, which polymerizes to form microtubules""
RPA135 SGDID:S000006214, Chr XVI from 581196-577585, Genome Release 64-1-1, reverse complement, Verified ORF, ""RNA polymerase I second largest subunit A135""
POR1 SGDID:S000005000, Chr XIV from 518845-517994, Genome Release 64-1-1, reverse complement, Verified ORF, ""Mitochondrial porin (voltage-dependent anion channel), outer membrane protein required for the maintenance of mitochondrial osmotic stabilit
RPL13B SGDID:S000004750, Chr XIII from 550801-550206,551207-551204, Genome Release 64-1-1, reverse complement, Verified ORF, ""Protein component of the large (60S) ribosomal subunit, nearly identical to Rpl13Ap; not essential for viability; has simil
MDM38 SGDID:S000005387, Chr XV from 273724-272003, Genome Release 64-1-1, reverse complement, Verified ORF, ""Mitochondrial protein, forms a complex with Mba1p to facilitate recruitment of mRNA-specific translational activators to ribosomes; roles in
PET9 SGDID:S000000126, Chr II from 163997-163041, Genome Release 64-1-1, reverse complement, Verified ORF, ""Major ADP/ATP carrier of the mitochondrial inner membrane, exchanges cytosolic ADP for mitochondrially synthesized ATP; also imports heme and
IDH1 SGDID:S000004982, Chr XIV from 559002-557920, Genome Release 64-1-1, reverse complement, Verified ORF, ""Subunit of mitochondrial NAD(+)-dependent isocitrate dehydrogenase, which catalyzes the oxidation of isocitrate to alpha-ketoglutarate in th
LYS20 SGDID:S000002341, Chr IV from 133437-134723, Genome Release 64-1-1, Verified ORF, ""Homocitrate synthase isozyme, catalyzes the condensation of acetyl-CoA and alpha-ketoglutarate to form homocitrate, which is the first step in the lysine biosyn
SSC1 SGDID:S000003806, Chr X from 521602-519638, Genome Release 64-1-1, reverse complement, Verified ORF, ""Hsp70 family ATPase, constituent of the import motor component of the Translocase of the Inner Mitochondrial membrane (TIM23 complex); involve
PUF6 SGDID:S000002904, Chr IV from 1443403-1441433, Genome Release 64-1-1, reverse complement, Verified ORF, ""Pumilio-homology domain protein that binds the 3' UTR of ASH1 mRNA and represses its translation, resulting in proper asymmetric localizati
RPL19B SGDID:S000000123, Chr II from 168423-168424,168809-169376, Genome Release 64-1-1, Verified ORF, ""Protein component of the large (60S) ribosomal subunit, nearly identical to Rpl19Ap and has similarity to rat L19 ribosomal protein; rpl19a and r
PSA1 SGDID:S000002213, Chr IV from 356759-355674, Genome Release 64-1-1, reverse complement, Verified ORF, ""GDP-mannose pyrophosphorylase (mannose-1-phosphate guanyltransferase), synthesizes GDP-mannose from GTP and mannose-1-phosphate in cell wall
NOC2 SGDID:S000005732, Chr XV from 727512-729644, Genome Release 64-1-1, Verified ORF, ""Protein that forms a nucleolar complex with Mak21p that binds to 90S and 66S pre-ribosomes, as well as a nuclear complex with Noc3p that binds to 66S pre-ribosom
HAS1 SGDID:S000004903, Chr XIII from 851591-850074, Genome Release 64-1-1, reverse complement, Verified ORF, ""ATP-dependent RNA helicase; localizes to both the nuclear periphery and nucleolus; highly enriched in nuclear pore complex fractions; const
YHC1 SGDID:S000004289, Chr XII from 725416-724721, Genome Release 64-1-1, reverse complement, Verified ORF, ""Component of the U1 snRNP complex required for pre-mRNA splicing; putative ortholog of human U1C protein, which is involved in formation of
SEA4 SGDID:S000000200, Chr II from 21293-18177, Genome Release 64-1-1, reverse complement, Verified ORF, ""Subunit of the SEA (Seh1-associated) complex, a coatomer-related complex that associates dynamically with the vacuole; has an N-terminal beta-p
URA7 SGDID:S000000135, Chr II from 145728-143989, Genome Release 64-1-1, reverse complement, Verified ORF, ""Major CTP synthase isozyme (see also URA8), catalyzes the ATP-dependent transfer of the amide nitrogen from glutamine to UTP, forming CTP, th
NPR2 SGDID:S000000788, Chr V from 34407-36254, Genome Release 64-1-1, Verified ORF, ""Subunit of the conserved Npr2/3 complex that mediates downregulation of TORC1 activity upon amino acid limitation; subunit of SEA (Seh1-associated) complex; homolog
HRR25 SGDID:S000006125, Chr XVI from 164276-165760, Genome Release 64-1-1, Verified ORF, ""Protein kinase involved in regulating diverse events including vesicular trafficking, DNA repair, and chromosome segregation; binds the CTD of RNA pol II; homo
RPL8A SGDID:S000001025, Chr VIII from 36025-35255, Genome Release 64-1-1, reverse complement, Verified ORF, ""Ribosomal protein L4 of the large (60S) ribosomal subunit, nearly identical to Rpl8Bp and has similarity to rat L7a ribosomal protein; mutat
RPT3 SGDID:S000002802, Chr IV from 1261681-1262967, Genome Release 64-1-1, Verified ORF, ""One of six ATPases of the 19S regulatory particle of the 26S proteasome involved in the degradation of ubiquitinated substrates; substrate of N-acetyltransfera
GCD11 SGDID:S000000827, Chr V from 205251-206834, Genome Release 64-1-1, Verified ORF, ""Gamma subunit of the translation initiation factor eIF2, involved in the identification of the start codon; binds GTP when forming the ternary complex with GTP a
STM1 SGDID:S000004140, Chr XII from 440467-441288, Genome Release 64-1-1, Verified ORF, ""Protein required for optimal translation under nutrient stress; perturbs association of Yef3p with ribosomes; involved in TOR signaling; binds G4 quadruplex and
TPI1 SGDID:S000002457, Chr IV from 556472-555726, Genome Release 64-1-1, reverse complement, Verified ORF, ""Triose phosphate isomerase, abundant glycolytic enzyme; mRNA half-life is regulated by iron availability; transcription is controlled by acti
BMS1 SGDID:S000006138, Chr XVI from 143171-139620, Genome Release 64-1-1, reverse complement, Verified ORF, ""GTPase required for synthesis of 40S ribosomal subunits and for processing the 35S pre-rRNA at sites A0, A1, and A2; interacts with Rcl1p, w
IQG1 SGDID:S000006163, Chr XVI from 95109-90622, Genome Release 64-1-1, reverse complement, Verified ORF, ""Essential protein required for determination of budding pattern, promotes localization of axial markers Bud4p and Cdc12p and functionally inte
NOP13 SGDID:S000005119, Chr XIV from 308612-307401, Genome Release 64-1-1, reverse complement, Verified ORF, ""Nucleolar protein found in preribosomal complexes; contains an RNA recognition motif (RRM)""
RPN10 SGDID:S000001243, Chr VIII from 499079-499885, Genome Release 64-1-1, Verified ORF, ""Non-ATPase base subunit of the 19S regulatory particle (RP) of the 26S proteasome; N-terminus plays a role in maintaining the structural integrity of the RP;
WSC3 SGDID:S000005465, Chr XV from 115808-114138, Genome Release 64-1-1, reverse complement, Verified ORF, ""Partially redundant sensor-transducer of the stress-activated PKC1-MPK1 signaling pathway involved in maintenance of cell wall integrity; inv
UTP9 SGDID:S000001239, Chr VIII from 491931-493658, Genome Release 64-1-1, Verified ORF, ""Nucleolar protein, component of the small subunit (SSU) processome containing the U3 snoRNA that is involved in processing of pre-18S rRNA""
COP1 SGDID:S000002304, Chr IV from 198176-194571, Genome Release 64-1-1, reverse complement, Verified ORF, ""Alpha subunit of COPI vesicle coatomer complex, which surrounds transport vesicles in the early secretory pathway""
FMP52 SGDID:S000000806, Chr V from 159580-160275, Genome Release 64-1-1, Verified ORF, ""Protein of unknown function, localized to the mitochondrial outer membrane; induced by treatment with 8-methoxypsoralen and UVA irradiation""
YTM1 SGDID:S000005798, Chr XV from 832813-834195, Genome Release 64-1-1, Verified ORF, ""Constituent of 66S pre-ribosomal particles, forms a complex with Nop7p and Erb1p that is required for maturation of the large ribosomal subunit; has seven C-term
SEC21 SGDID:S000005231, Chr XIV from 91994-94801, Genome Release 64-1-1, Verified ORF, ""Gamma subunit of coatomer, a heptameric protein complex that together with Arf1p forms the COPI coat; involved in ER to Golgi transport of selective cargo""
HMG1 SGDID:S000004540, Chr XIII from 118898-115734, Genome Release 64-1-1, reverse complement, Verified ORF, ""One of two isozymes of HMG-CoA reductase that catalyzes the conversion of HMG-CoA to mevalonate, which is a rate-limiting step in sterol bi
GFD2 SGDID:S000000541, Chr III from 59026-60726, Genome Release 64-1-1, Verified ORF, ""Protein of unknown function, identified as a high-copy suppressor of a dbp5 mutation""
NIP7 SGDID:S000006132, Chr XVI from 153495-154040, Genome Release 64-1-1, Verified ORF, ""Nucleolar protein required for 60S ribosome subunit biogenesis, constituent of 66S pre-ribosomal particles; physically interacts with Nop8p and the exosome subu
DBP8 SGDID:S000001212, Chr VIII from 442181-443476, Genome Release 64-1-1, Verified ORF, ""ATPase, putative RNA helicase of the DEAD-box family; component of 90S preribosome complex involved in production of 18S rRNA and assembly of 40S small ribosom
CKA1 SGDID:S000001297, Chr IX from 288908-287790, Genome Release 64-1-1, reverse complement, Verified ORF, ""Alpha catalytic subunit of casein kinase 2 (CK2), a Ser/Thr protein kinase with roles in cell growth and proliferation; CK2, comprised of CKA
YMR086W SGDID:S000004692, Chr XIII from 439208-442090, Genome Release 64-1-1, Verified ORF, ""Component of the eisosome with unknown function; may interact with ribosomes, based on co-purification experiments; GFP-fusion protein localizes to the cell
RPO31 SGDID:S000005642, Chr XV from 544145-539763, Genome Release 64-1-1, reverse complement, Verified ORF, ""RNA polymerase III largest subunit C160, part of core enzyme; similar to bacterial beta-prime subunit and to RPA190 and RPO21""
TMA17 SGDID:S000002268, Chr IV from 264964-264512, Genome Release 64-1-1, reverse complement, Verified ORF, ""Protein of unknown function that associates with ribosomes; heterozygous deletion demonstrated increases in chromosome instability in a rad9
YCF1 SGDID:S000002542, Chr IV from 727551-723004, Genome Release 64-1-1, reverse complement, Verified ORF, ""Vacuolar glutathione S-conjugate transporter of the ATP-binding cassette family, has a role in detoxifying metals such as cadmium, mercury, a
FMP27 SGDID:S000004446, Chr XII from 1043998-1051884, Genome Release 64-1-1, Uncharacterized ORF, ""Putative protein of unknown function; the authentic, non-tagged protein is detected in highly purified mitochondria in high-throughput studies""
CCW14 SGDID:S000006429, Chr XII from 903724-904440, Genome Release 64-1-1, Verified ORF, ""Covalently linked cell wall glycoprotein, present in the inner layer of the cell wall""
MDR1 SGDID:S000003332, Chr VII from 690245-693097, Genome Release 64-1-1, Verified ORF, ""Cytoplasmic GTPase-activating protein for Ypt/Rab transport GTPases Ypt6p, Ypt31p and Sec4p; involved in recycling of internalized proteins and regulation of Go
HFA1 SGDID:S000004820, Chr XIII from 683564-677193, Genome Release 64-1-1, reverse complement, Verified ORF, ""Mitochondrial acetyl-coenzyme A carboxylase, catalyzes the production of malonyl-CoA in mitochondrial fatty acid biosynthesis""
SSN2 SGDID:S000002851, Chr IV from 1349938-1345676, Genome Release 64-1-1, reverse complement, Verified ORF, ""Subunit of the RNA polymerase II mediator complex; associates with core polymerase subunits to form the RNA polymerase II holoenzyme; requi
MRPL15 SGDID:S000004304, Chr XII from 759480-760241, Genome Release 64-1-1, Verified ORF, ""Mitochondrial ribosomal protein of the large subunit""
RTN2 SGDID:S000002363, Chr IV from 94605-95786, Genome Release 64-1-1, Verified ORF, ""Protein of unknown function; has similarity to mammalian reticulon proteins; member of the RTNLA (reticulon-like A) subfamily""
ASH1 SGDID:S000001668, Chr XI from 94499-96265, Genome Release 64-1-1, Verified ORF, ""Zinc-finger inhibitor of HO transcription; mRNA is localized and translated in the distal tip of anaphase cells, resulting in accumulation of Ash1p in daughter cel
RPB2 SGDID:S000005677, Chr XV from 616671-612997, Genome Release 64-1-1, reverse complement, Verified ORF, ""RNA polymerase II second largest subunit B150, part of central core; similar to bacterial beta subunit""
ICE2 SGDID:S000001352, Chr IX from 193595-195070, Genome Release 64-1-1, Verified ORF, ""Integral ER membrane protein with type-III transmembrane domains; mutations cause defects in cortical ER morphology in both the mother and daughter cells""
LTE1 SGDID:S000000022, Chr I from 105872-101565, Genome Release 64-1-1, reverse complement, Verified ORF, ""Protein similar to GDP/GTP exchange factors but without detectable GEF activity; required for asymmetric localization of Bfa1p at daughter-dir
DSN1 SGDID:S000001449, Chr IX from 375431-377161, Genome Release 64-1-1, Verified ORF, ""Essential component of the MIND kinetochore complex (Mtw1p Including Nnf1p-Nsl1p-Dsn1p) which joins kinetochore subunits contacting DNA to those contacting micro
MTC5 SGDID:S000002535, Chr IV from 709549-712995, Genome Release 64-1-1, Verified ORF, ""Subunit of the SEA (Seh1-associated) complex, a coatomer-related complex that associates dynamically with the vacuole; has N-terminal WD-40 repeats and a C-termi
EST1 SGDID:S000004223, Chr XII from 609524-607425, Genome Release 64-1-1, reverse complement, Verified ORF, ""TLC1 RNA-associated factor involved in telomere length regulation as recruitment subunit of telomerase; has G-quadruplex promoting activity
NAB3 SGDID:S000006111, Chr XVI from 187725-185317, Genome Release 64-1-1, reverse complement, Verified ORF, ""Single stranded RNA binding protein; acidic ribonucleoprotein; required for termination of non-poly(A) transcripts and efficient splicing; i

Supplemental Table S2. Pkc1-associated proteins without HU only

**Description**
RPS9A SGDID:S000006002, Chr XVI from 404950-404956,405458-406044, Genome Release 64-1-1, Verified ORF, ""Protein component of the small (40S) ribosomal subunit; nearly identical to Rps9Bp and has similarity to E. coli S4 and rat S9 ribosomal proteins
RPL2B SGDID:S000001280, Chr IX from 316768-316771,317172-317932, Genome Release 64-1-1, Verified ORF, ""Protein component of the large (60S) ribosomal subunit, identical to Rpl2Ap and has similarity to E. coli L2 and rat L8 ribosomal proteins; expres
PET10 SGDID:S000001754, Chr XI from 525074-524223, Genome Release 64-1-1, reverse complement, Verified ORF, ""Protein of unknown function that co-purifies with lipid particles; expression pattern suggests a role in respiratory growth; computational a
RPL27B SGDID:S000002879, Chr IV from 1401770-1401800,1402185-1402564, Genome Release 64-1-1, Verified ORF, ""Protein component of the large (60S) ribosomal subunit, nearly identical to Rpl27Ap and has similarity to rat L27 ribosomal protein""
RPL13A SGDID:S000002240, Chr IV from 308424-308427,308793-309388, Genome Release 64-1-1, Verified ORF, ""Protein component of the large (60S) ribosomal subunit, nearly identical to Rpl13Bp; not essential for viability; has similarity to rat L13 ribos
YRA1 SGDID:S000002789, Chr IV from 1236558-1236842,1237609-1238004, Genome Release 64-1-1, Verified ORF, ""RNA binding protein required for export of poly(A)+ mRNA from the nucleus; proposed to couple mRNA export with 3' end processing via its intera
RPL8B SGDID:S000003968, Chr XII from 48629-47859, Genome Release 64-1-1, reverse complement, Verified ORF, ""Ribosomal protein L4 of the large (60S) ribosomal subunit, nearly identical to Rpl8Ap and has similarity to rat L7a ribosomal protein; mutati
RPS2 SGDID:S000003091, Chr VII from 277617-278381, Genome Release 64-1-1, Verified ORF, ""Protein component of the small (40S) subunit, essential for control of translational accuracy; phosphorylation by C-terminal domain kinase I (CTDK-I) enhances t
ENO1 SGDID:S000003486, Chr VIII from 1000927-1002240, Genome Release 64-1-1, Verified ORF, ""Enolase I, a phosphopyruvate hydratase that catalyzes the conversion of 2-phosphoglycerate to phosphoenolpyruvate during glycolysis and the reverse reaction d
RPS1B SGDID:S000004528, Chr XIII from 146482-147249, Genome Release 64-1-1, Verified ORF, ""Ribosomal protein 10 (rp10) of the small (40S) subunit; nearly identical to Rps1Ap and has similarity to rat S3a ribosomal protein""
HTA2 SGDID:S000000099, Chr II from 235792-235394, Genome Release 64-1-1, reverse complement, Verified ORF, ""Histone H2A, core histone protein required for chromatin assembly and chromosome function; one of two nearly identical (see also HTA1) subtyp
YGR161C-D SGDID:S000007368, Chr VII from 821709-817747,823015-821711, Genome Release 64-1-1, reverse complement, transposable_element_gene, ""Retrotransposon TYA Gag and TYB Pol genes; transcribed/translated as one unit; polyprotein is processed to m
RPS31 SGDID:S000004157, Chr XII from 498947-499405, Genome Release 64-1-1, Verified ORF, ""Fusion protein that is cleaved to yield a ribosomal protein of the small (40S) subunit and ubiquitin; ubiquitin may facilitate assembly of the ribosomal protei
ERG27 SGDID:S000004090, Chr XII from 341810-342853, Genome Release 64-1-1, Verified ORF, ""3-keto sterol reductase, catalyzes the last of three steps required to remove two C-4 methyl groups from an intermediate in ergosterol biosynthesis; mutants ar
ZRT1 SGDID:S000003224, Chr VII from 20978-22108, Genome Release 64-1-1, Verified ORF, ""High-affinity zinc transporter of the plasma membrane, responsible for the majority of zinc uptake; transcription is induced under low-zinc conditions by the Zap1
DPM1 SGDID:S000006387, Chr XVI from 900755-901558, Genome Release 64-1-1, Verified ORF, ""Dolichol phosphate mannose (Dol-P-Man) synthase of the ER membrane, catalyzes the formation of Dol-P-Man from Dol-P and GDP-Man; required for glycosyl phosphati
EPL1 SGDID:S000001870, Chr VI from 90345-87847, Genome Release 64-1-1, reverse complement, Verified ORF, ""Component of NuA4, which is an essential histone H4/H2A acetyltransferase complex; homologous to Drosophila Enhancer of Polycomb""
RPS8A SGDID:S000000168, Chr II from 89125-88523, Genome Release 64-1-1, reverse complement, Verified ORF, ""Protein component of the small (40S) ribosomal subunit; identical to Rps8Bp and has similarity to rat S8 ribosomal protein""
RPL21B SGDID:S000006000, Chr XVI from 406636-406646,407068-407539, Genome Release 64-1-1, Verified ORF, ""Protein component of the large (60S) ribosomal subunit, nearly identical to Rpl21Ap and has similarity to rat L21 ribosomal protein""
ERG1 SGDID:S000003407, Chr VII from 848423-846933, Genome Release 64-1-1, reverse complement, Verified ORF, ""Squalene epoxidase, catalyzes the epoxidation of squalene to 2,3-oxidosqualene; plays an essential role in the ergosterol-biosynthesis pathw
KGD1 SGDID:S000001387, Chr IX from 122689-125733, Genome Release 64-1-1, Verified ORF, ""Component of the mitochondrial alpha-ketoglutarate dehydrogenase complex, which catalyzes a key step in the tricarboxylic acid (TCA) cycle, the oxidative decarbo
RPS27B SGDID:S000001063, Chr VIII from 148116-147871,148669-148667, Genome Release 64-1-1, reverse complement, Verified ORF, ""Protein component of the small (40S) ribosomal subunit; nearly identical to Rps27Ap and has similarity to rat S27 ribosomal
SEF1 SGDID:S000000162, Chr II from 100115-96669, Genome Release 64-1-1, reverse complement, Verified ORF, ""Putative transcription factor, has homolog in Kluyveromyces lactis""
RPL43A SGDID:S000006247, Chr XVI from 654166-654167,654571-654847, Genome Release 64-1-1, Verified ORF, ""Protein component of the large (60S) ribosomal subunit, identical to Rpl43Bp and has similarity to rat L37a ribosomal protein; null mutation con
SAC7 SGDID:S000002797, Chr IV from 1252537-1254501, Genome Release 64-1-1, Verified ORF, ""GTPase activating protein (GAP) for Rho1p, involved in signaling to the actin cytoskeleton, null mutations suppress tor2 mutations and temperature sensitive mu
PAT1 SGDID:S000000673, Chr III from 252628-250238, Genome Release 64-1-1, reverse complement, Verified ORF, ""Topoisomerase II-associated deadenylation-dependent mRNA-decapping factor; also required for faithful chromosome transmission, maintenance o
MSS116 SGDID:S000002602, Chr IV from 847946-845952, Genome Release 64-1-1, reverse complement, Verified ORF, ""DEAD-box protein required for efficient splicing of mitochondrial Group I and II introns; non-polar RNA helicase that also facilities stran
SGF73 SGDID:S000003034, Chr VII from 377609-379582, Genome Release 64-1-1, Verified ORF, ""SAGA complex subunit; has a role in anchoring the deubiquitination module into SAGA and SLIK complexes; involved in preinitiation complex assembly at promoters
RPS17A SGDID:S000004486, Chr XIII from 225889-225891,226290-226697, Genome Release 64-1-1, Verified ORF, ""Ribosomal protein 51 (rp51) of the small (40s) subunit; nearly identical to Rps17Bp and has similarity to rat S17 ribosomal protein""
HSC82 SGDID:S000004798, Chr XIII from 632355-634472, Genome Release 64-1-1, Verified ORF, ""Cytoplasmic chaperone of the Hsp90 family, redundant in function and nearly identical with Hsp82p, and together they are essential; expressed constitutively a
CDC48 SGDID:S000002284, Chr IV from 238664-236157, Genome Release 64-1-1, reverse complement, Verified ORF, ""ATPase involved in ubiquitin-mediated protein degradation; Cdc48p-Npl4p-Ufd1p complex participates in ER-associated degradation (ERAD) while
SLS1 SGDID:S000004129, Chr XII from 423473-421542, Genome Release 64-1-1, reverse complement, Verified ORF, ""Mitochondrial membrane protein that coordinates expression of mitochondrially-encoded genes; may facilitate delivery of mRNA to membrane-bou
OSH6 SGDID:S000001711, Chr XI from 445381-446727, Genome Release 64-1-1, Verified ORF, ""Member of an oxysterol-binding protein family with overlapping, redundant functions in sterol metabolism and which collectively perform a function essential for
SSO1 SGDID:S000006153, Chr XVI from 107275-108147, Genome Release 64-1-1, Verified ORF, ""Plasma membrane t-SNARE involved in fusion of secretory vesicles at the plasma membrane and in vesicle fusion during sporulation; forms a complex with Sec9p tha
ROG1 SGDID:S000003112, Chr VII from 234507-232450, Genome Release 64-1-1, reverse complement, Verified ORF, ""Protein with putative serine active lipase domain""
MSH6 SGDID:S000002504, Chr IV from 643837-640109, Genome Release 64-1-1, reverse complement, Verified ORF, ""Protein required for mismatch repair in mitosis and meiosis, forms a complex with Msh2p to repair both single-base & insertion-deletion mispa
THO2 SGDID:S000005083, Chr XIV from 365717-360924, Genome Release 64-1-1, reverse complement, Verified ORF, ""Subunit of the THO complex, which is required for efficient transcription elongation and involved in transcriptional elongation-associated r
YMR185W SGDID:S000004797, Chr XIII from 629025-631970, Genome Release 64-1-1, Uncharacterized ORF, ""Putative protein of unknown function; conflicting evidence on whether null mutant is viable with elongated buds, or inviable""

Supplemental Table S3. SILAC mass spectrometry of Pkc1 phosphorylation sites, HU treated versus untreated.

(gl) Pyro-glu
(ac) acetylation
(ox) oxidation
(de) deamidation
(ph) phosphorylation

peptide	start	phospho number	phospho sites	protein	silac01 - Ratio H/L (untreated vs. HU)	silac02 - Ratio H/L (untreated vs. HU)	silac03 - Ratio H/L (untreated vs. HU)
(ac)S(ph)FSQLEQNIK	2	1	2S	PKC1	0.40	0.43	0.30
(ac)SFSQLEQNIKK	2	0		PKC1	0.79		1.27
(ac)SFSQLEQNIK	2	0		PKC1	0.83	0.82	
KIAVEENIIR	13	0		PKC1	0.91		
IAVEENIIR	14	0		PKC1			0.99
KKTSNVM(ox)VIQK	29	0		PKC1		0.82	
KKTSNVMVIQK	29	0		PKC1		0.85	1.17
KTSNVM(ox)VIQK	30	0		PKC1	0.83	0.76	
KTSNVMVIQK	30	0		PKC1	0.81	0.83	
TSNVM(ox)VIQK	31	0		PKC1	0.85	0.86	
TSNVMVIQK	31	0		PKC1	0.82	0.87	
QNLEYLEDSLKK	49	0		PKC1	0.84	0.84	1.17
(gl)QNLEYLEDSLKK	49	0		PKC1	0.86		1.08
(gl)QNLEYLEDSLK	49	0		PKC1	0.88	0.92	
QNLEYLEDSLK	49	0		PKC1	0.90	0.86	
EYGFLSTK	85	0		PKC1	0.82	0.87	1.27
IQYM(ox)LQQLEFK	116	0		PKC1	0.82	1.08	1.71
IQYMLQQLEFK	116	0		PKC1	0.83	0.88	1.15
(gl)QYQEANTK	132	0		PKC1	0.85		
SSSAAEGGAMESK	152	0		PKC1	0.86	0.80	1.13
SSSAAEGGAM(ox)ESK	152	0		PKC1	0.85		
KYQAINVDFDQFK	176	0		PKC1	0.80	0.86	0.54
YQAINVDFDQFK	177	0		PKC1	0.82		1.27
HQPN(de)DIM(ox)DNQQPK	189	0		PKC1	0.81	0.86	
HQPNDIM(ox)DNQQPK	189	0		PKC1	0.93	0.87	
HQPNDIMDNQQPK	189	0		PKC1	0.88	0.89	1.76
RKQLTGVLTIGITAAR	204	0		PKC1			1.15
DVDHIQSPM(ox)FAR	220	0		PKC1	0.89	0.98	1.48
KPESYVTIK	232	0		PKC1	0.82	0.82	1.20
WSEDFQIPVEK	257	0		PKC1	2.62	0.91	1.29
GNEIEITVYDK	268	0		PKC1	0.96	1.50	
QLMGGLHR	391	0		PKC1			1.25
(gl)QLM(ox)GGLHR	391	0		PKC1	0.76	0.92	
KEEIFEQHGHHFVQK	406	0		PKC1	0.81	0.88	1.23
EEIFEQHGHHFVQK	407	0		PKC1	0.89	0.83	1.21
YTGFQCQDCK	437	0		PKC1	0.91		
KCYTNVVTK	452	0		PKC1	1.14		
CYTNVVTK	453	0		PKC1	0.78		
TSTDTDPDEAK	465	0		PKC1	0.89		1.40
TVPSAQLGSSIGTANGSDLS(ph)PSKLAER	558	1	577S	PKC1	0.85		0.75
TVPSAQLGSSIGTAN(de)GSDLS(ph)PSK	558	1	577S	PKC1	0.80	0.63	
TVPSAQLGSSIGTANGSDLS(ph)PSK	558	1	577S	PKC1	0.77	0.61	
TVPSAQLGSSIGTAN(de)GSDLS(ph)PSKLAER	558	1	577S	PKC1	0.78	0.44	
TVPSAQLGSSIGTAN(de)GSDLSPSK	558	0		PKC1	1.00	3.54	4.46
TVPSAQLGSSIGTANGSDLSPSK	558	0		PKC1	1.04		
ANAPLPPQPR	585	0		PKC1	0.98		1.57
KHDKT(ph)PS(ph)PQK	595	2	599T / 601S	PKC1			0.62
KHDKTPS(ph)PQK	595	1	601S	PKC1	0.89	0.83	1.12
KHDKTPSPQK	595	0		PKC1	1.04	3.09	4.30
VGRDS(ph)PTKQHDPIIDK	605	1	609S	PKC1	1.00		
DSPTKQHDPIIDKR	608	0		PKC1		3.75	3.21
QHDPIIDK	613	0		PKC1	1.17		
(gl)QHDPIIDK	613	0		PKC1	1.07		
FIDENEAYLN(de)FTEGAQQTAEFSS(ph)PEK	635	1	657S	PKC1	0.55		
FIDENEAYLNFTEGAQQTAEFSS(ph)PEK	635	1	657S	PKC1	0.76	0.52	0.69
FIDENEAYLNFTEGAQQTAEFSSPEK	635	0		PKC1	1.08		
TLDPTSNR	661	0		PKC1	0.96		
TLDPTSN(de)RR	661	0		PKC1	0.87		
S(ph)LGLTDLSIEHSQTWESK	670	1	670S	PKC1	0.83		1.62
SLGLTDLSIEHSQTWESK	670	0		PKC1	1.21		
AQREEM(ox)ELEIKQDSGEIQEDLEVDHIDLETK	700	0		PKC1		0.90	
EEMELEIK	703	0		PKC1	0.90		
QDSGEIQEDLEVDHIDLETK	711	0		PKC1	0.89		
(gl)QDSGEIQEDLEVDHIDLETK	711	0		PKC1	0.87		
(gl)QKLDWENK	731	0		PKC1	0.97	1.02	
QKLDWENKN(de)DFR	731	0		PKC1	0.89		
QKLDWENK	731	0		PKC1	0.87	0.98	
(gl)EADLTIDSTHTNPFR	743	0		PKC1	7.48		
DM(ox)NS(ph)ETFQIEQDHASK	758	1	761S	PKC1	1.03		
DMNS(ph)ETFQIEQDHASK	758	1	761S	PKC1	0.88		
DMN(de)SETFQIEQDHASK	758	0		PKC1	0.80		
DM(ox)N(de)SETFQIEQDHASK	758	0		PKC1	0.87		
DMNSETFQIEQDHASK	758	0		PKC1	0.89	0.97	
DM(ox)NSETFQIEQDHASK	758	0		PKC1	0.95	0.99	
DMNSETFQIEQDHASKEVLQETVSLAPTSTHASR	758	0		PKC1			1.81
EVLQET(ph)VSLAPTSTHASR	774	1	779T	PKC1	1.07		1.18
EVLQETVS(ph)LAPTSTHASR	774	1	781S	PKC1	0.98	1.18	
TTDQQS(ph)PQKSQTSTSAK	792	1	797S	PKC1	0.81	0.52	0.79
TTDQQS(ph)PQKS(ph)QTSTSAK	792	2	797S / 801S	PKC1	0.85		
KVS(ph)LDNFVLLK	818	1	820S	PKC1	0.94		1.73
KVSLDNFVLLK	818	0		PKC1	0.92	1.06	0.94
VSLDN(de)FVLLK	819	0		PKC1	0.97	1.06	
VSLDNFVLLK	819	0		PKC1	0.89	1.03	1.12
KDNIIQNHDIESARAEK	857	0		PKC1		0.60	
KDNIIQN(de)HDIESAR	857	0		PKC1	0.98		26.96
DNIIQNHDIESAR	858	0		PKC1	0.84	1.11	
DNIIQN(de)HDIESAR	858	0		PKC1	0.91		
DNIIQ(de)NHDIESAR	858	0		PKC1	1.01		1.23
DNIIQNHDIESARAEK	858	0		PKC1		0.83	3.59
KVFLLATK	874	0		PKC1	0.92	1.00	0.66
VFLLATK	875	0		PKC1	0.91		1.15
AKFYAAEVLLALK	926	0		PKC1	1.11	1.11	1.22

FYAAEVLLALK	928	0		PKC1	0.93	1.06	1.12
YFHDNGVIYR	939	0		PKC1	1.03		0.93
DLKLENILLTPEGHIK	949	0		PKC1	0.98	1.00	1.05
LENILLTPEGHIK	952	0		PKC1	0.94	0.97	1.16
T(ph)STFCGTPEFM(ox)APEILK	981	1	982S	PKC1	1.20		
T(ph)STFCGTPEFMAPEILKEQEYTK	981	1	983T	PKC1	0.94		
T(ph)STFCGTPEFM(ox)APEILKEQEYTK	981	1	983T	PKC1		0.92	
TST(ph)FCGTPEFMAPEILK	981	1	983T	PKC1	1.00		
TST(ph)FCGTPEFM(ox)APEILKEQEYTK	981	1	983T	PKC1	0.93		
TST(ph)FCGTPEFM(ox)APEILK	981	1	983T	PKC1	0.98	1.05	
DADEVM(ox)EEPFFR	1073	0		PKC1		1.19	
NIN(de)FDDILNLR	1085	0		PKC1	0.98		
VKPPYIPEIK	1096	0		PKC1	1.02	1.03	1.25
PPYIPEIK	1098	0		PKC1	0.74	0.89	
_SPEDTSYFEQEFTSAPPT(ph)LTPLPSVLTTSQQEEFF	1106	1	1125T	PKC1		1.21	
GFSFM(ox)PDDLDLSGSAAR	1141	0		PKC1		0.92	
AADITS(ph)LYK	1161	1	1166S	PKC1	1.43	1.03	0.78
AADITSLYKIK	1161	0		PKC1		1.11	
AADITSLYK	1161	0		PKC1	1.00	1.18	1.16
TAALAQHDEAAGK	1224	0		PKC1	1.04	1.06	1.04
TAALAQHDEAAGKAGEGEIPAPLAGTVSK	1224	0		PKC1	1.18	1.17	1.17
AGEGEIPAPLAGTVSK	1237	0		PKC1	0.99	1.08	1.18
ILVKEGDTVK	1253	0		PKC1	1.11	1.14	1.07
DAVQGGQGLIK	1296	0		PKC1	1.10	1.11	1.12
